# Supplementary material for: An alternative mechanism by which If1 prevents ATP hydrolysis by the ATP synthase subcomplex in S. cerevisiae
Source: EMBO Rep. 2025 Jun 9;26(13):3305–26. doi: 10.1038/s44319-025-00430-8 (PMC12238618; doi:10.1038/s44319-025-00430-8)
Supplement: Supplementary file 1 — Table EV1 [file 44319_2025_430_MOESM1_ESM.docx]

Table EV1 Table listing the oligonucleotides used for strain construction.
