# Supplementary figures and images for: An alternative mechanism by which If1 prevents ATP hydrolysis by the ATP synthase subcomplex in S. cerevisiae (part 1 of 2)
Source: EMBO Rep. 2025 Jun 9;26(13):3305–26. doi: 10.1038/s44319-025-00430-8 (PMC12238618; doi:10.1038/s44319-025-00430-8)

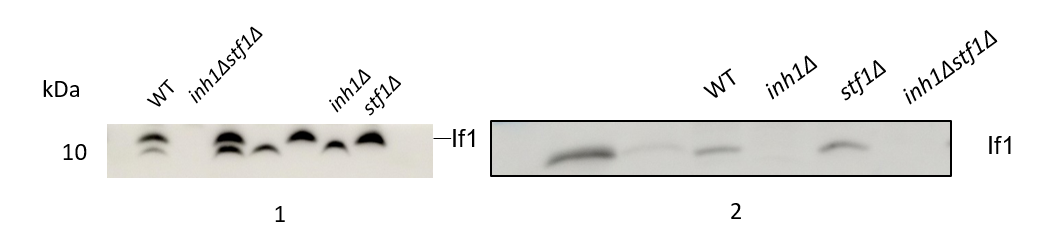

Supplement: Supplementary file 3 — Source data Fig. 1 [file 44319_2025_430_MOESM3_ESM.zip › Figure 1/1A/western If1 replicate.tif]

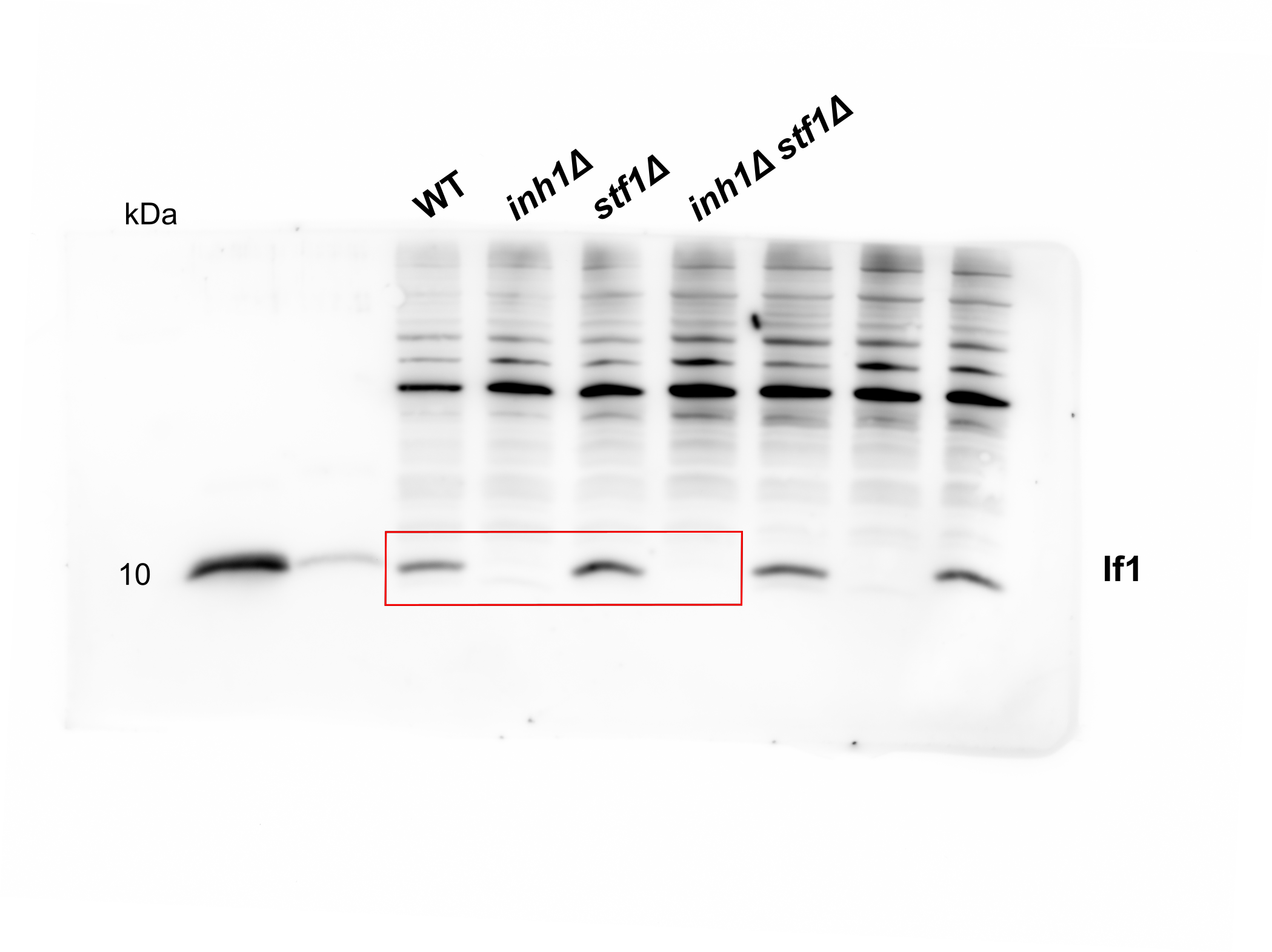

Supplement: Supplementary file 3 — Source data Fig. 1 [file 44319_2025_430_MOESM3_ESM.zip › Figure 1/1A/western If1.tiff]

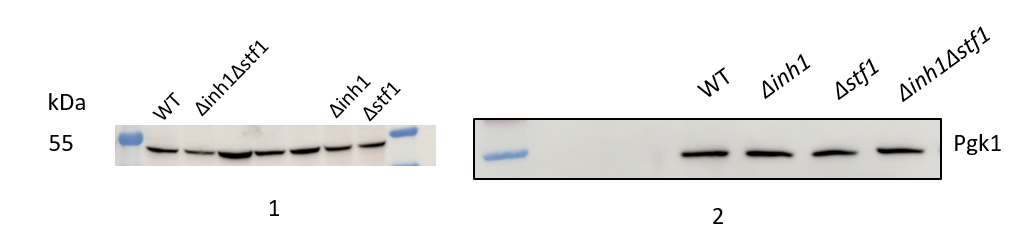

Supplement: Supplementary file 3 — Source data Fig. 1 [file 44319_2025_430_MOESM3_ESM.zip › Figure 1/1A/western Pgk1 replicate.tif]

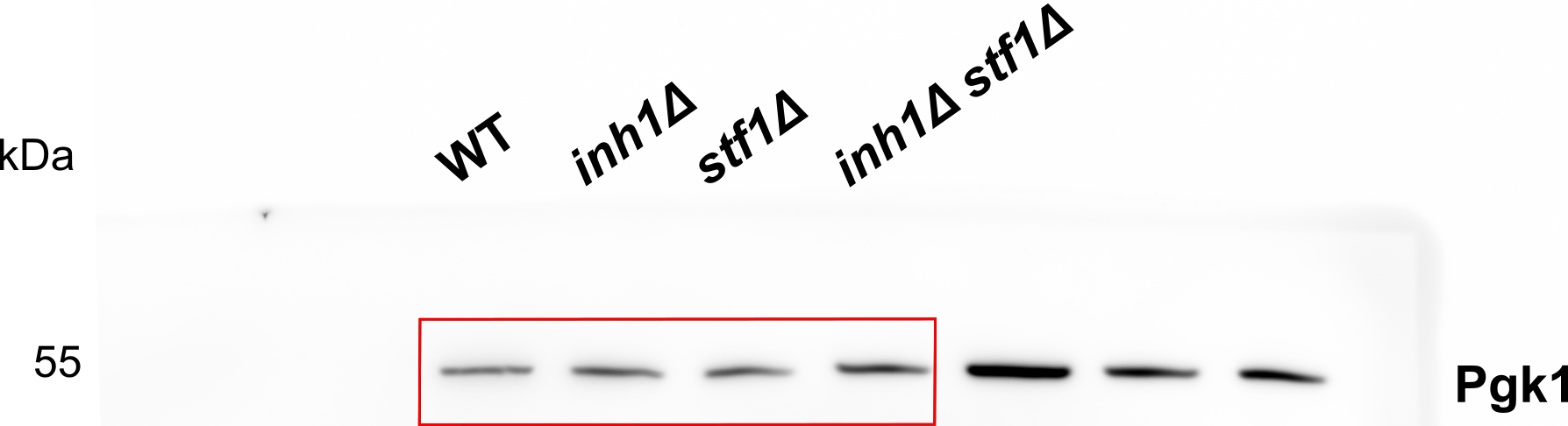

Supplement: Supplementary file 3 — Source data Fig. 1 [file 44319_2025_430_MOESM3_ESM.zip › Figure 1/1A/western Pgk1.tiff]

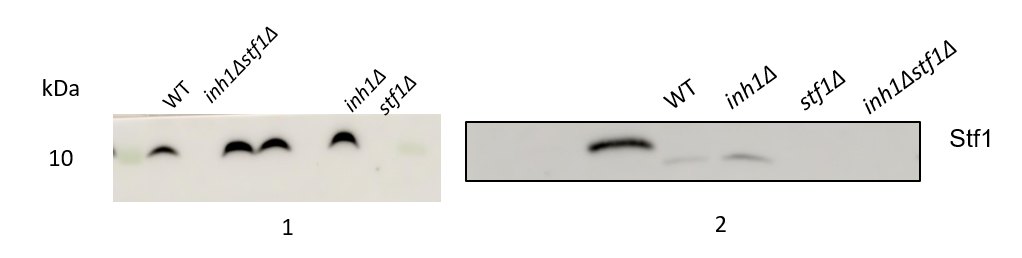

Supplement: Supplementary file 3 — Source data Fig. 1 [file 44319_2025_430_MOESM3_ESM.zip › Figure 1/1A/western Stf1 replicate.tif]

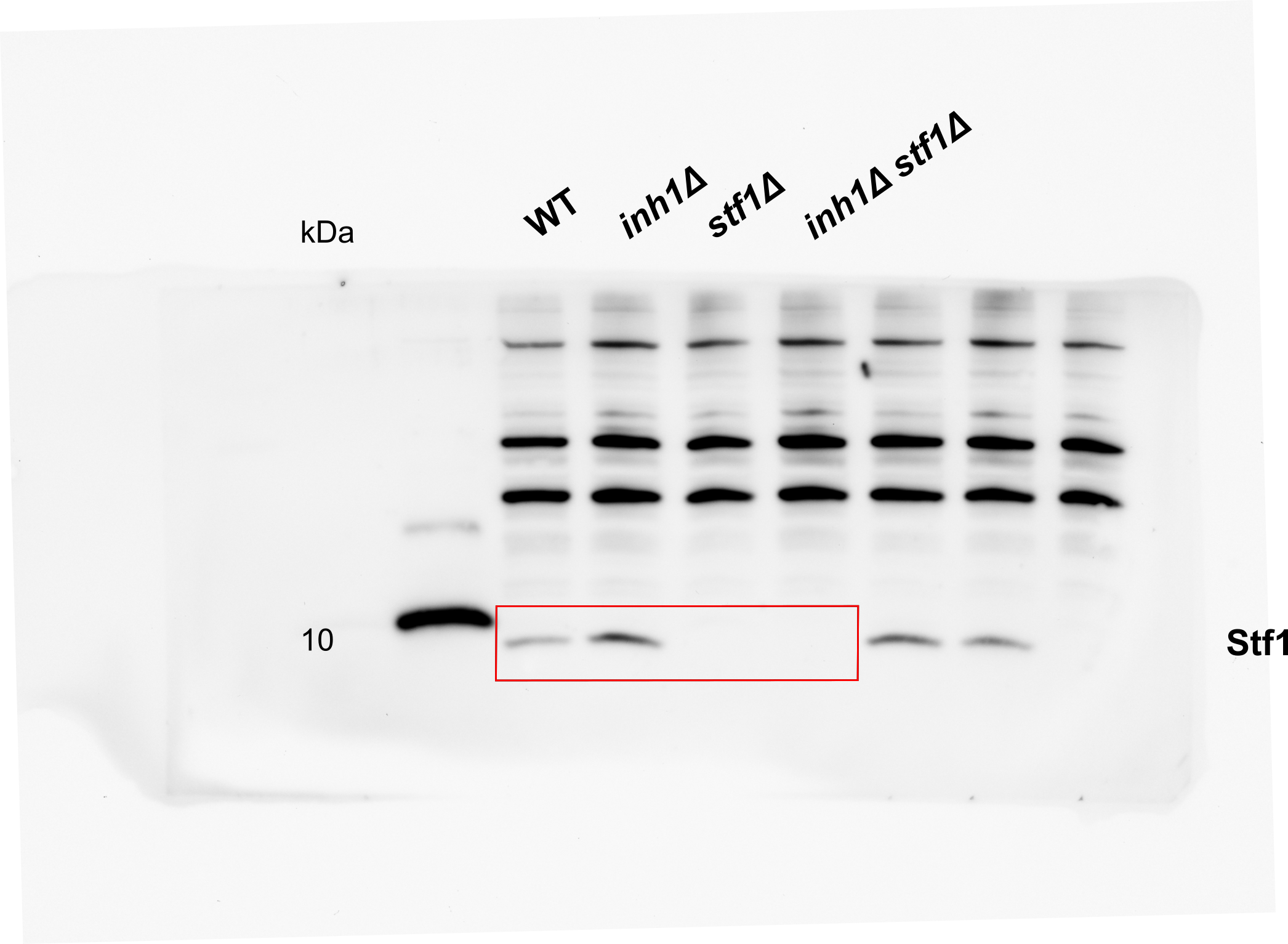

Supplement: Supplementary file 3 — Source data Fig. 1 [file 44319_2025_430_MOESM3_ESM.zip › Figure 1/1A/western Stf1.tiff]

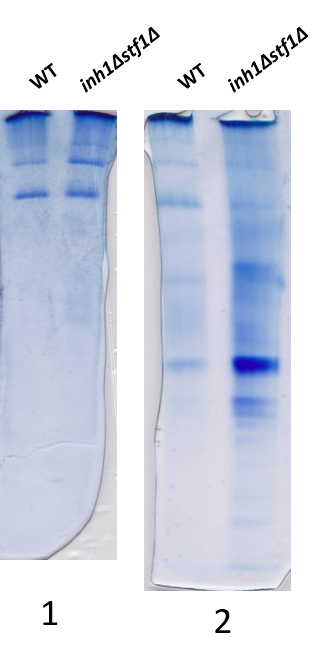

Supplement: Supplementary file 3 — Source data Fig. 1 [file 44319_2025_430_MOESM3_ESM.zip › Figure 1/1C/Coomassie replicate.tif]

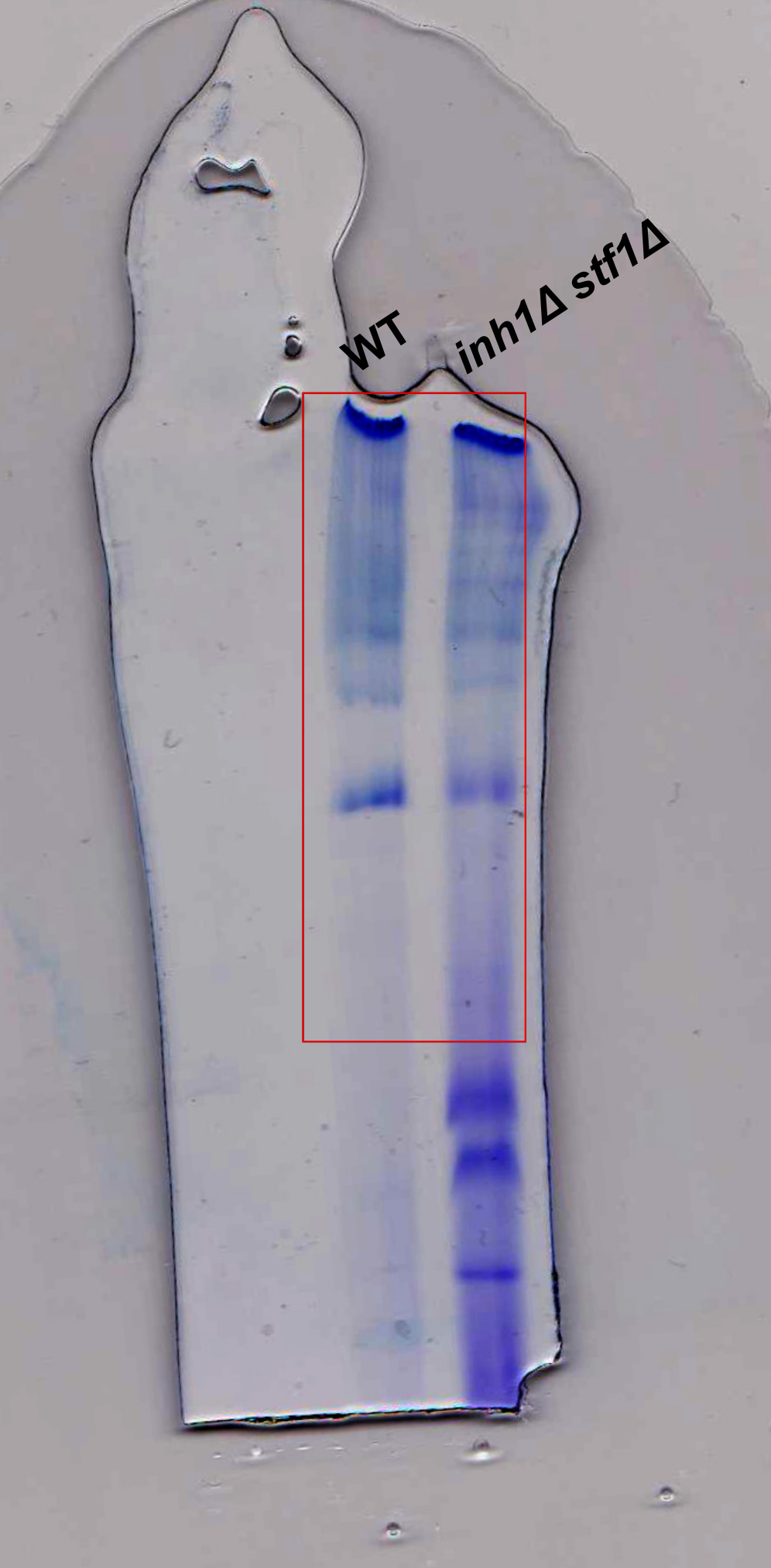

Supplement: Supplementary file 3 — Source data Fig. 1 [file 44319_2025_430_MOESM3_ESM.zip › Figure 1/1C/Coomassie.tiff]

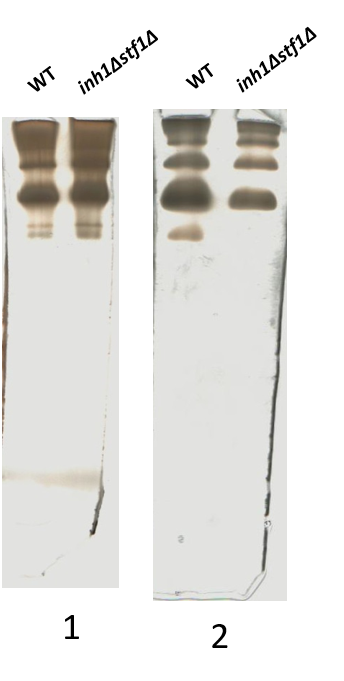

Supplement: Supplementary file 3 — Source data Fig. 1 [file 44319_2025_430_MOESM3_ESM.zip › Figure 1/1C/CV in gel activity replicate.tif]

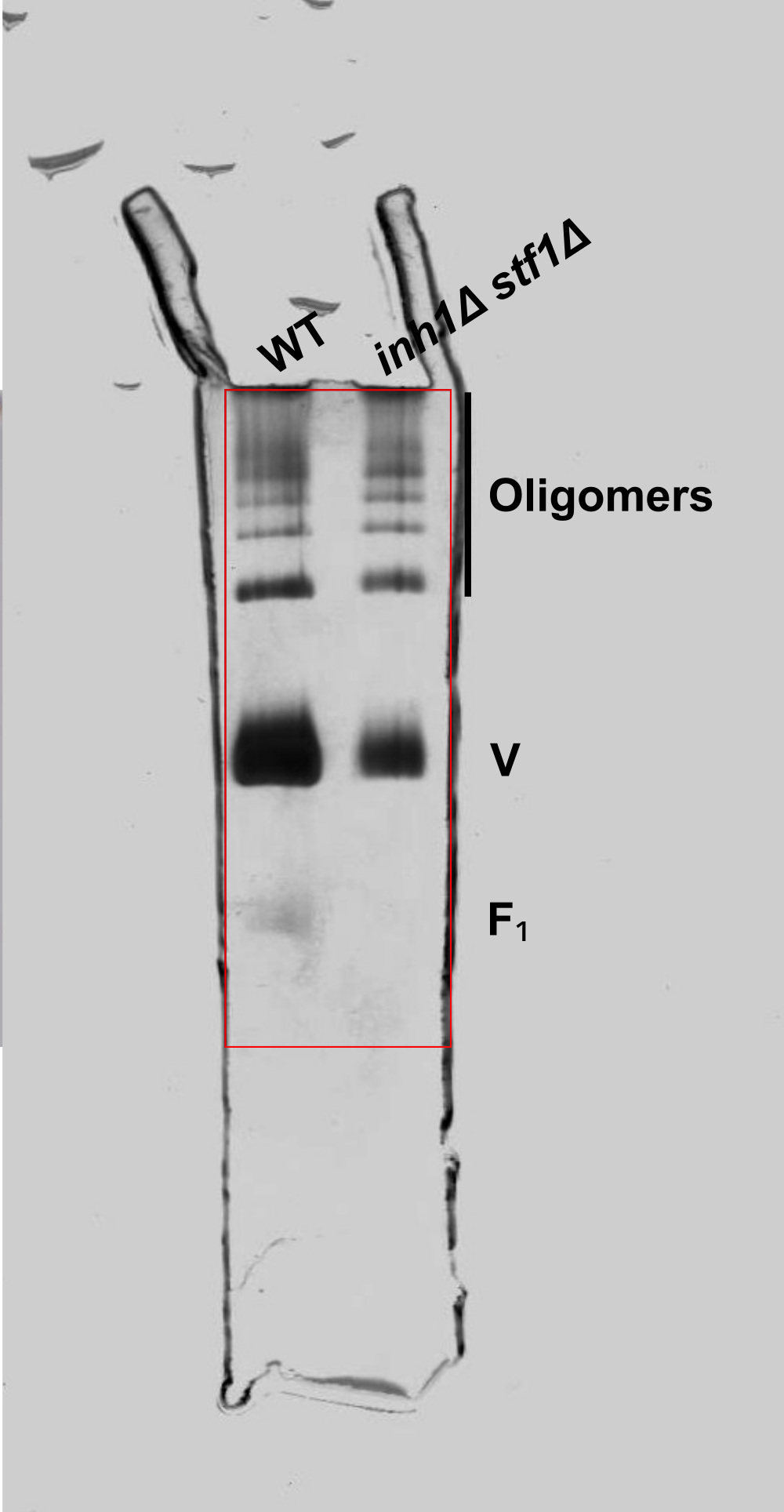

Supplement: Supplementary file 3 — Source data Fig. 1 [file 44319_2025_430_MOESM3_ESM.zip › Figure 1/1C/CV in gel activity.tiff]

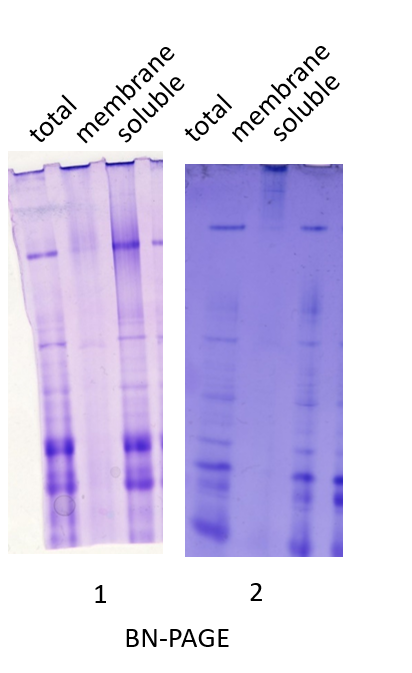

Supplement: Supplementary file 3 — Source data Fig. 1 [file 44319_2025_430_MOESM3_ESM.zip › Figure 1/1D/Coomassie BN-PAGE replicate.tif]

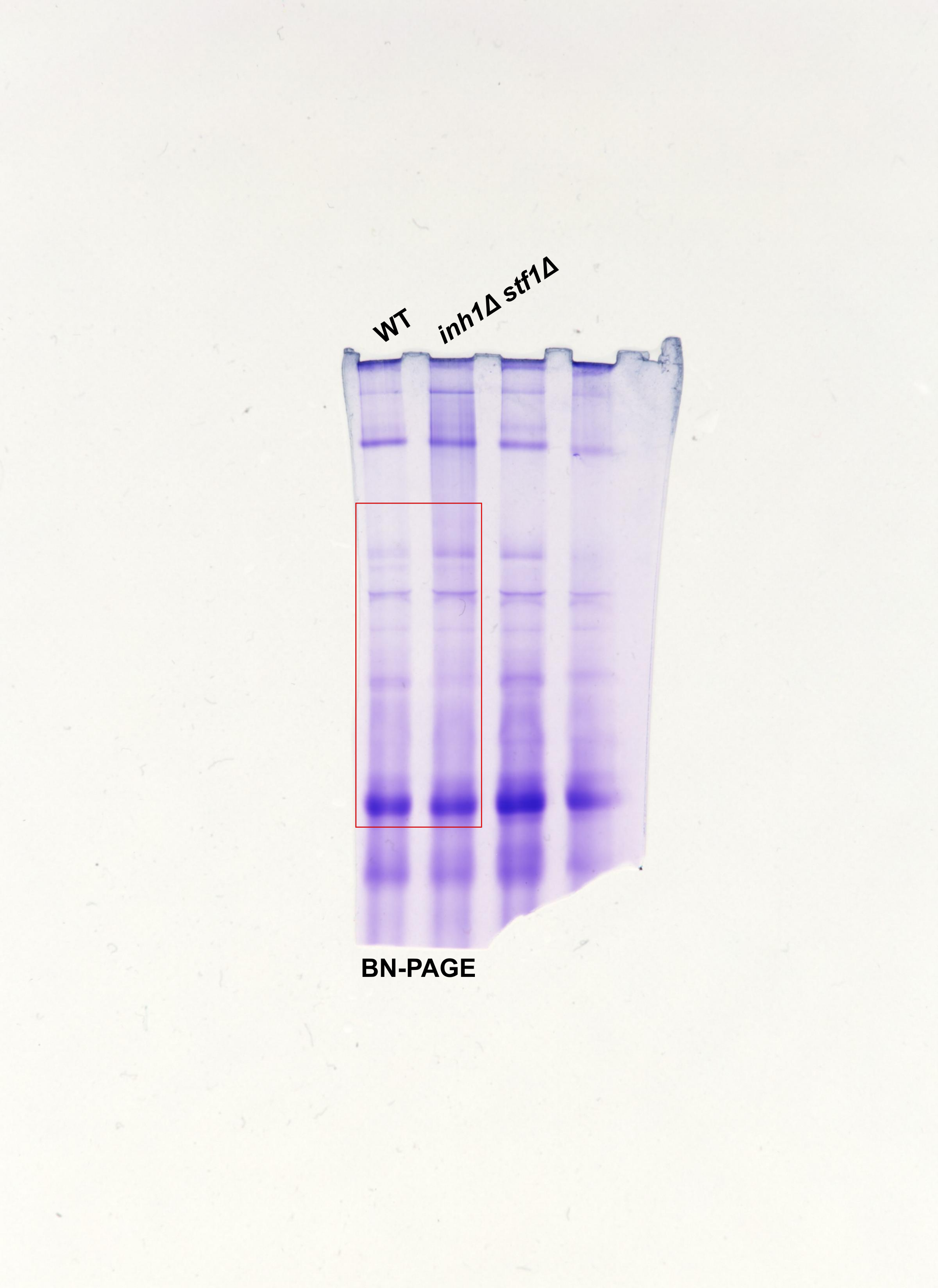

Supplement: Supplementary file 3 — Source data Fig. 1 [file 44319_2025_430_MOESM3_ESM.zip › Figure 1/1D/Coomassie BN-PAGE.tiff]

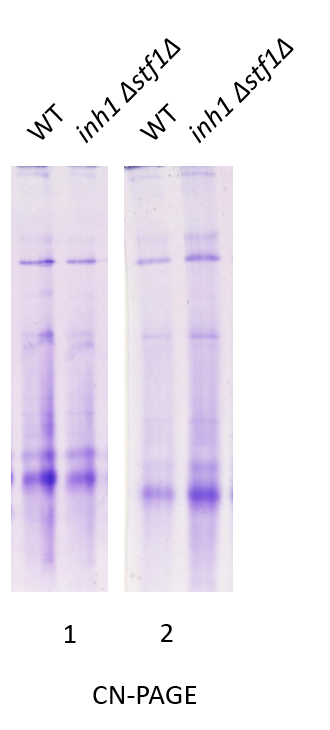

Supplement: Supplementary file 3 — Source data Fig. 1 [file 44319_2025_430_MOESM3_ESM.zip › Figure 1/1D/Coomassie CN-PAGE replicate.tif]

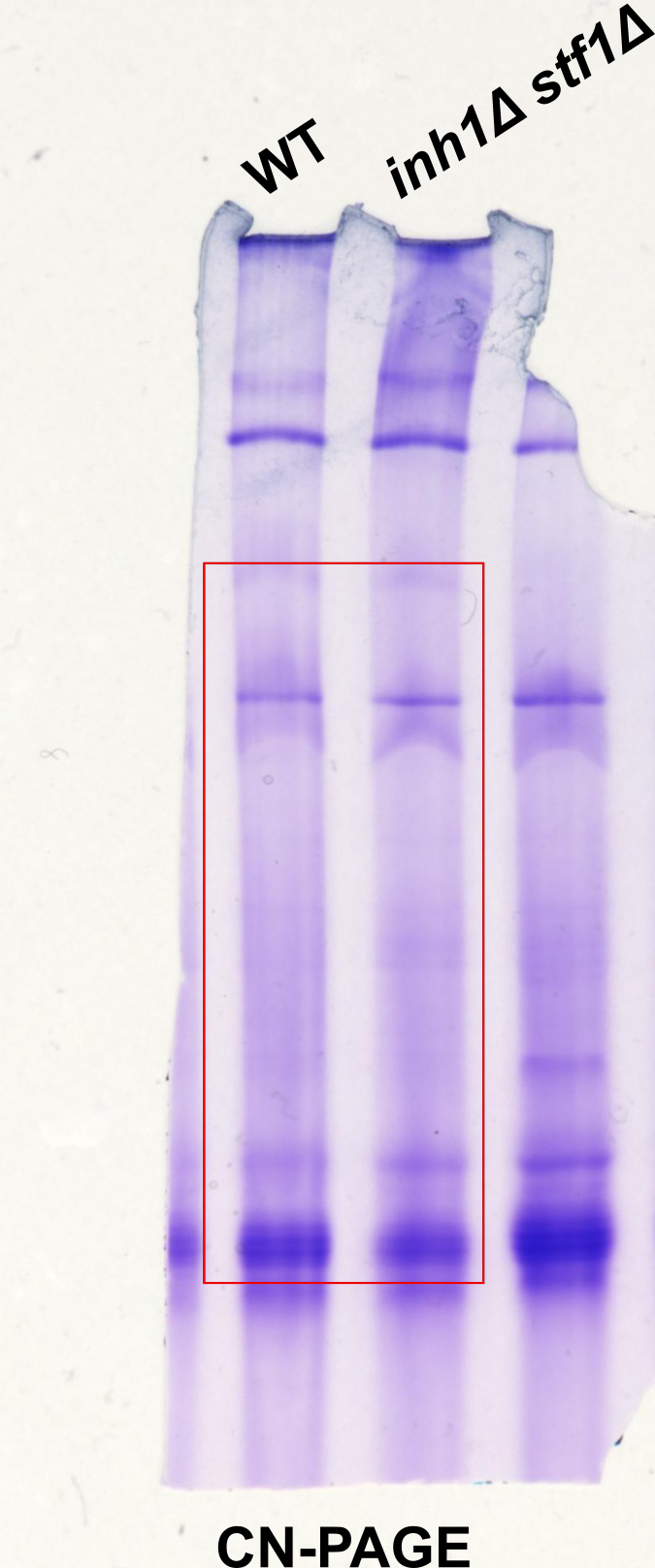

Supplement: Supplementary file 3 — Source data Fig. 1 [file 44319_2025_430_MOESM3_ESM.zip › Figure 1/1D/Coomassie CN-PAGE.tiff]

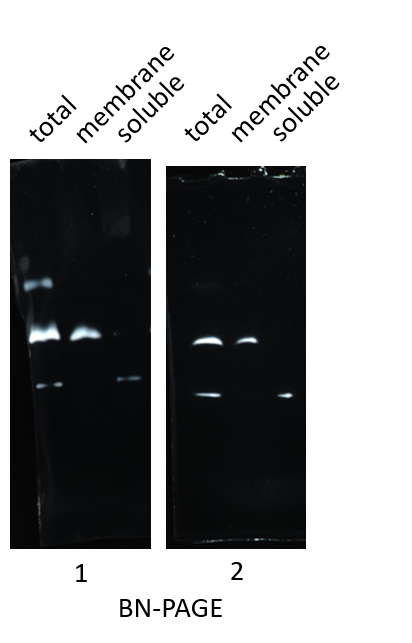

Supplement: Supplementary file 3 — Source data Fig. 1 [file 44319_2025_430_MOESM3_ESM.zip › Figure 1/1D/CV in gel activity BN-PAGE replicate.tif]

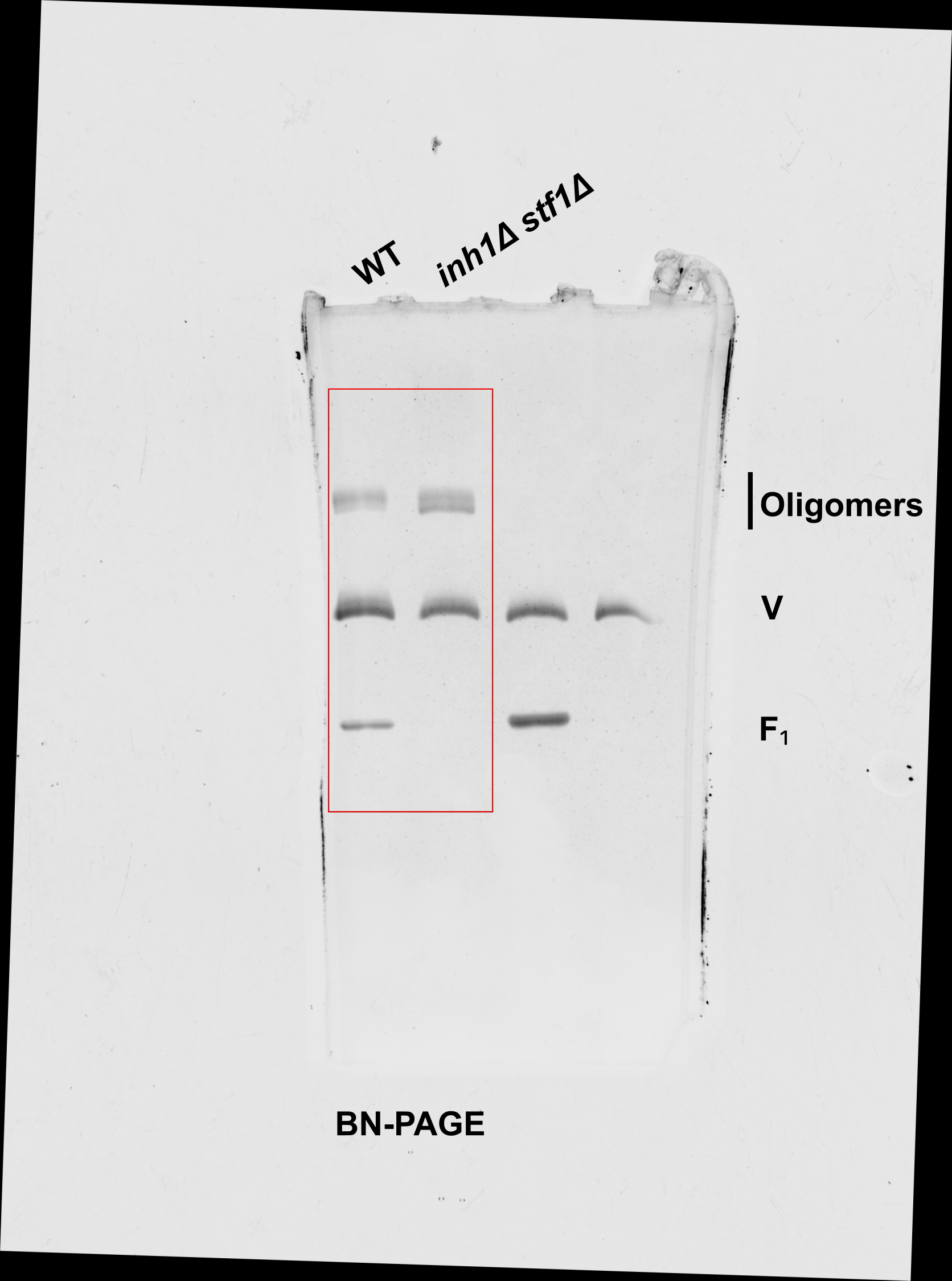

Supplement: Supplementary file 3 — Source data Fig. 1 [file 44319_2025_430_MOESM3_ESM.zip › Figure 1/1D/CV in gel activity BN-PAGE.tiff]

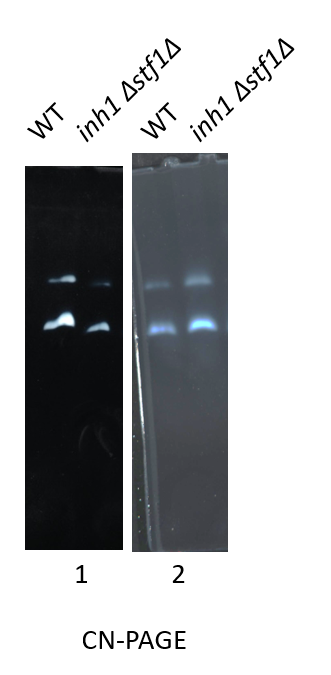

Supplement: Supplementary file 3 — Source data Fig. 1 [file 44319_2025_430_MOESM3_ESM.zip › Figure 1/1D/CV in gel activity CN-PAGE replicate.tif]

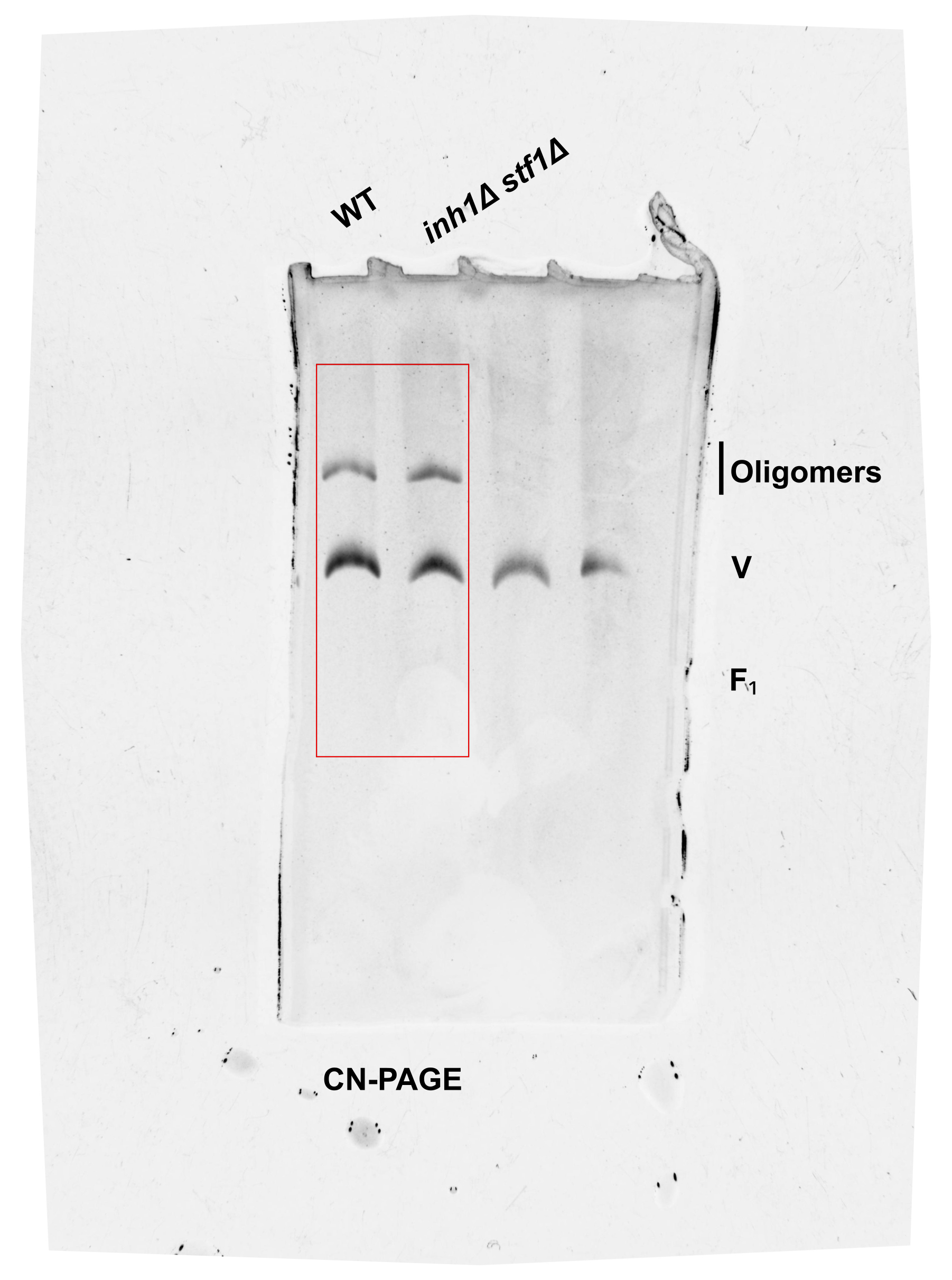

Supplement: Supplementary file 3 — Source data Fig. 1 [file 44319_2025_430_MOESM3_ESM.zip › Figure 1/1D/CV in gel activity CN-PAGE.tiff]

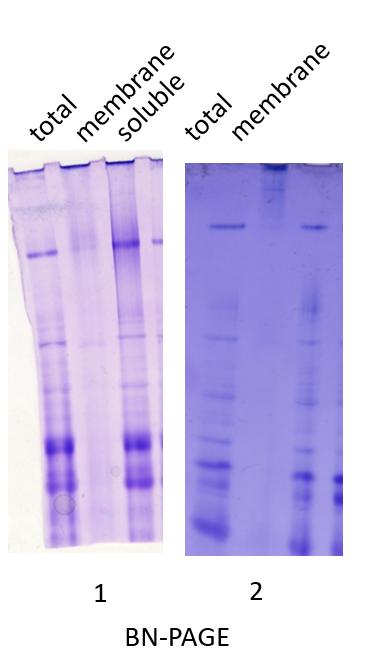

Supplement: Supplementary file 3 — Source data Fig. 1 [file 44319_2025_430_MOESM3_ESM.zip › Figure 1/1E/Coomassie BN-PAGE replicate.tif]

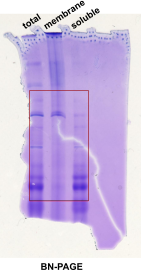

Supplement: Supplementary file 3 — Source data Fig. 1 [file 44319_2025_430_MOESM3_ESM.zip › Figure 1/1E/Coomassie BN-PAGE.tiff]

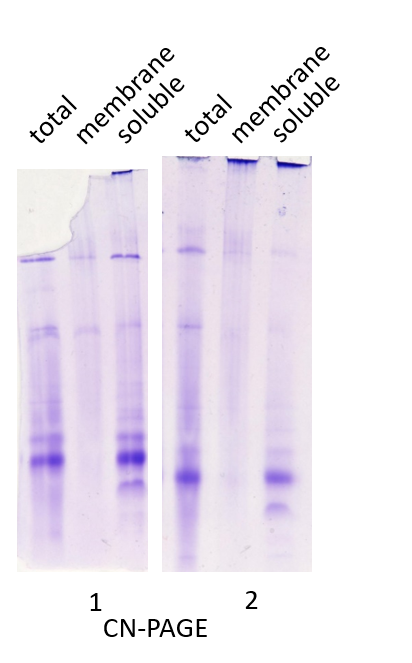

Supplement: Supplementary file 3 — Source data Fig. 1 [file 44319_2025_430_MOESM3_ESM.zip › Figure 1/1E/Coomassie CN-PAGE replicate.tif]

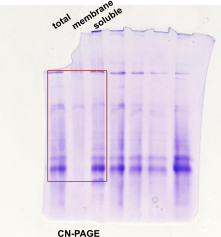

Supplement: Supplementary file 3 — Source data Fig. 1 [file 44319_2025_430_MOESM3_ESM.zip › Figure 1/1E/Coomassie CN-PAGE.tiff]

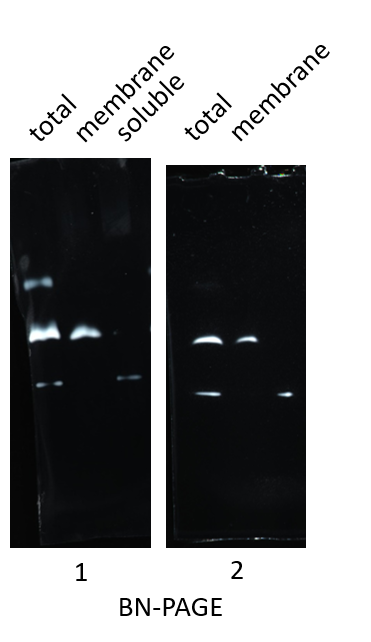

Supplement: Supplementary file 3 — Source data Fig. 1 [file 44319_2025_430_MOESM3_ESM.zip › Figure 1/1E/CV in gel activity BN-PAGE replicate.tif]

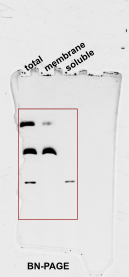

Supplement: Supplementary file 3 — Source data Fig. 1 [file 44319_2025_430_MOESM3_ESM.zip › Figure 1/1E/CV in gel activity BN-PAGE.tiff]

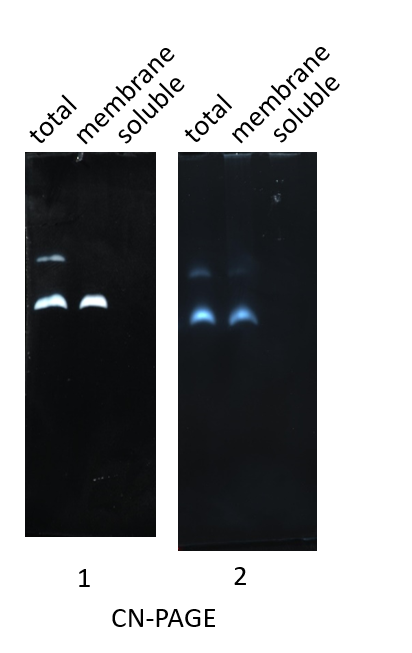

Supplement: Supplementary file 3 — Source data Fig. 1 [file 44319_2025_430_MOESM3_ESM.zip › Figure 1/1E/CV in gel activity CN-PAGE replicate.tif]

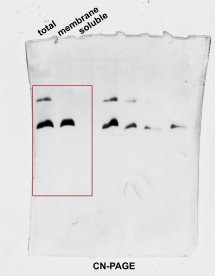

Supplement: Supplementary file 3 — Source data Fig. 1 [file 44319_2025_430_MOESM3_ESM.zip › Figure 1/1E/CV in gel activity CN-PAGE.tiff]

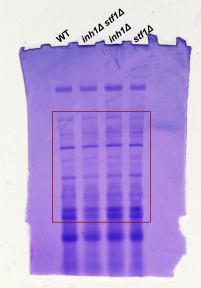

Supplement: Supplementary file 3 — Source data Fig. 1 [file 44319_2025_430_MOESM3_ESM.zip › Figure 1/1F/Coomassie.tiff]

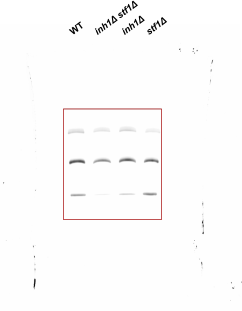

Supplement: Supplementary file 3 — Source data Fig. 1 [file 44319_2025_430_MOESM3_ESM.zip › Figure 1/1F/CV in gel activity.tiff]

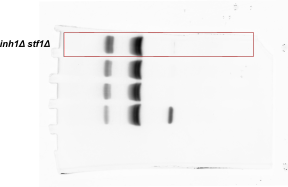

Supplement: Supplementary file 4 — Source data Fig. 2 [file 44319_2025_430_MOESM4_ESM.zip › Figure 2/2A/mutant/CV in gel activity.tiff]

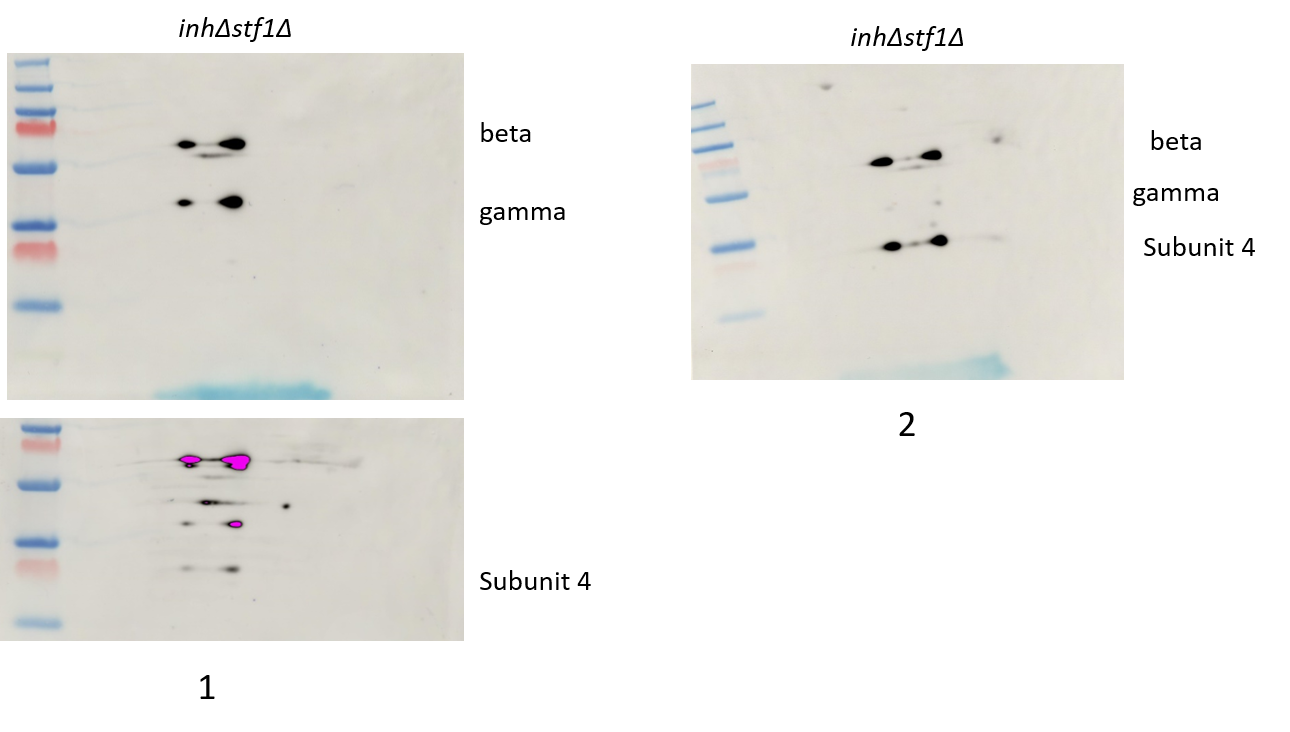

Supplement: Supplementary file 4 — Source data Fig. 2 [file 44319_2025_430_MOESM4_ESM.zip › Figure 2/2A/mutant/western beta gamma subunit 4 replicate.tif]

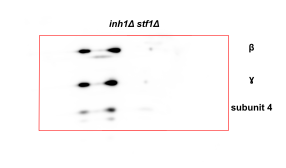

Supplement: Supplementary file 4 — Source data Fig. 2 [file 44319_2025_430_MOESM4_ESM.zip › Figure 2/2A/mutant/western beta gamma subunit4.tiff]

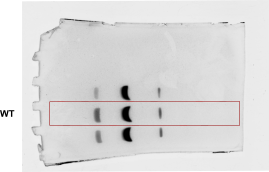

Supplement: Supplementary file 4 — Source data Fig. 2 [file 44319_2025_430_MOESM4_ESM.zip › Figure 2/2A/WT/1D WT IGA.tiff]

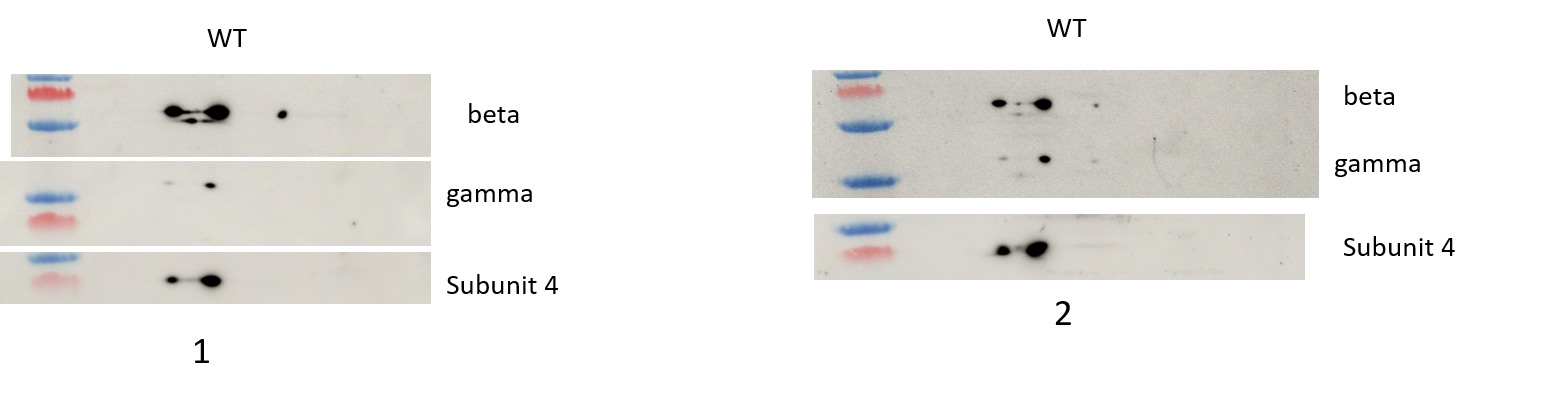

Supplement: Supplementary file 4 — Source data Fig. 2 [file 44319_2025_430_MOESM4_ESM.zip › Figure 2/2A/WT/western beta gamma subunit 4 replicate.tif]

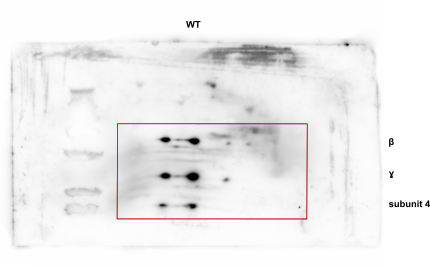

Supplement: Supplementary file 4 — Source data Fig. 2 [file 44319_2025_430_MOESM4_ESM.zip › Figure 2/2A/WT/western beta gamma subunit 4.tiff]

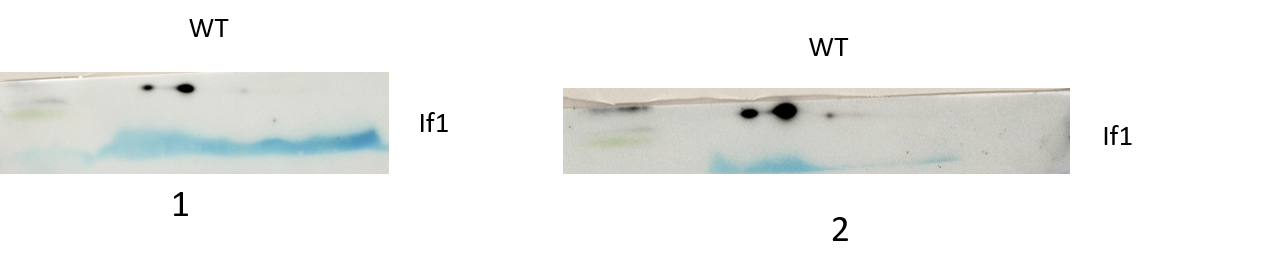

Supplement: Supplementary file 4 — Source data Fig. 2 [file 44319_2025_430_MOESM4_ESM.zip › Figure 2/2A/WT/western If1 replicate.tif]

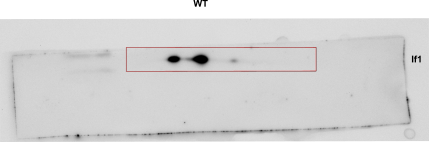

Supplement: Supplementary file 4 — Source data Fig. 2 [file 44319_2025_430_MOESM4_ESM.zip › Figure 2/2A/WT/western If1.tiff]

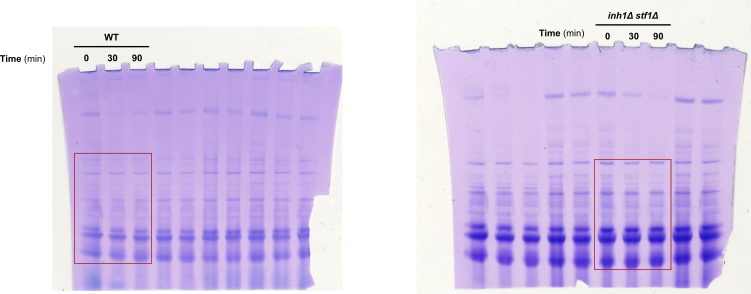

Supplement: Supplementary file 5 — Source data Fig. 3 [file 44319_2025_430_MOESM5_ESM.zip › Figure 3/3A/Coomassie.tiff]

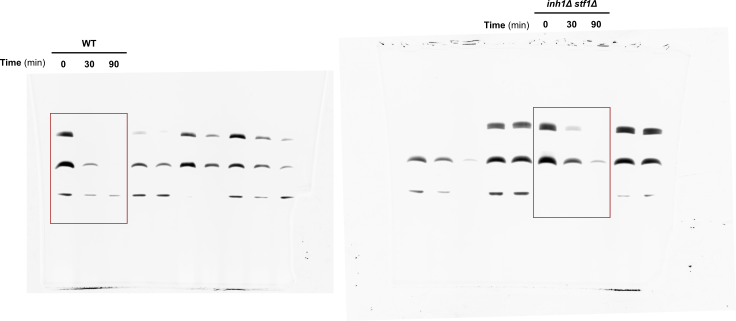

Supplement: Supplementary file 5 — Source data Fig. 3 [file 44319_2025_430_MOESM5_ESM.zip › Figure 3/3A/CV in gel activity.tiff]

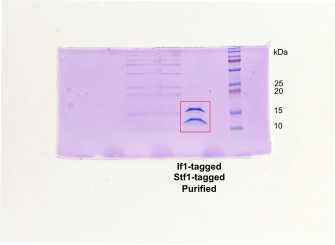

Supplement: Supplementary file 5 — Source data Fig. 3 [file 44319_2025_430_MOESM5_ESM.zip › Figure 3/3B/coomassie If1 Stf1 purified.tiff]

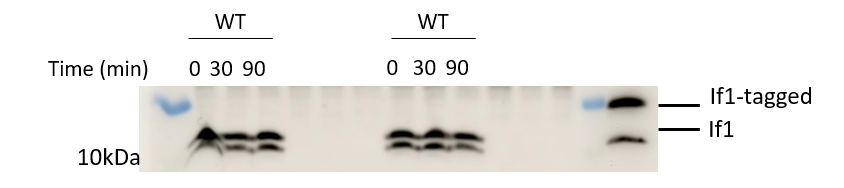

Supplement: Supplementary file 5 — Source data Fig. 3 [file 44319_2025_430_MOESM5_ESM.zip › Figure 3/3B/western If1 replicate.tif]

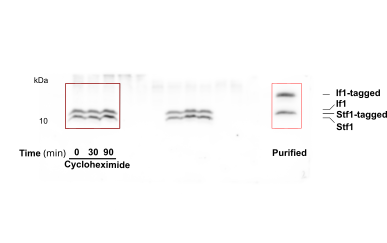

Supplement: Supplementary file 5 — Source data Fig. 3 [file 44319_2025_430_MOESM5_ESM.zip › Figure 3/3B/western if1 stf1.tiff]

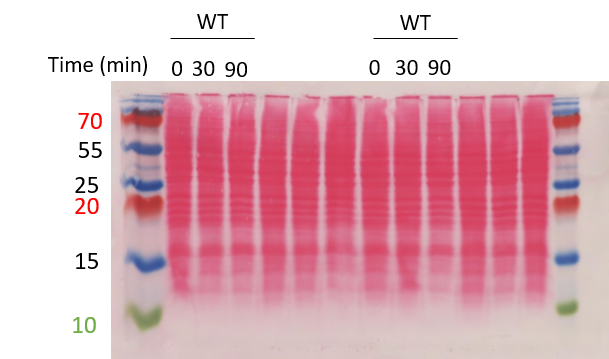

Supplement: Supplementary file 5 — Source data Fig. 3 [file 44319_2025_430_MOESM5_ESM.zip › Figure 3/3B/western ponceau replicate.tif]

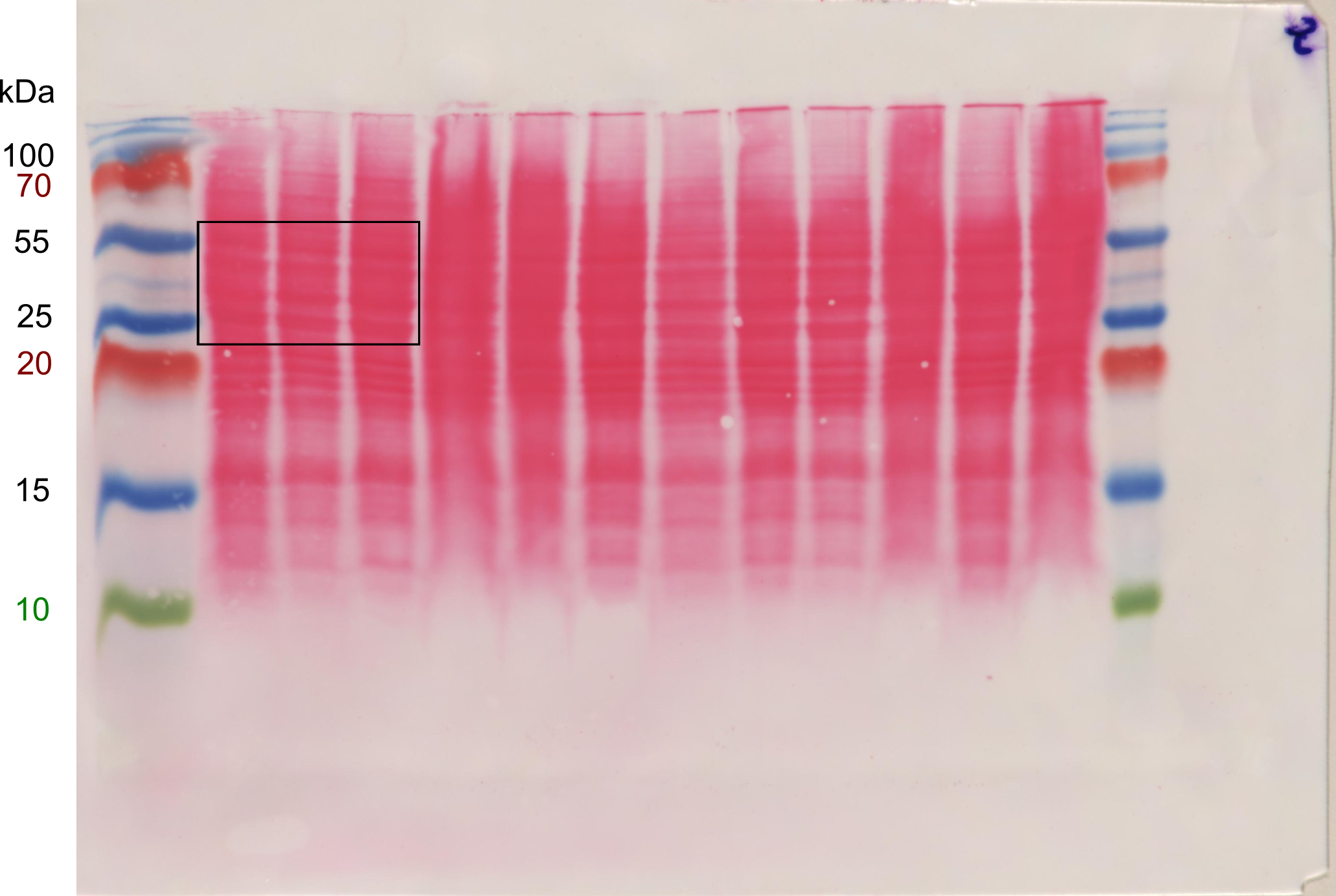

Supplement: Supplementary file 5 — Source data Fig. 3 [file 44319_2025_430_MOESM5_ESM.zip › Figure 3/3B/western ponceau.tiff]

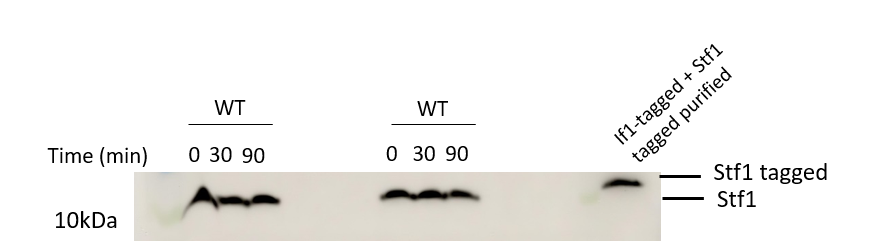

Supplement: Supplementary file 5 — Source data Fig. 3 [file 44319_2025_430_MOESM5_ESM.zip › Figure 3/3B/western Stf1 replicate.tif]

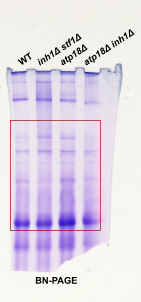

Supplement: Supplementary file 5 — Source data Fig. 3 [file 44319_2025_430_MOESM5_ESM.zip › Figure 3/3C/Coomassie BN-PAGE.tiff]

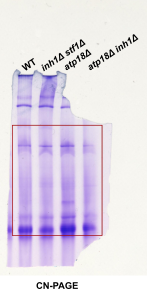

Supplement: Supplementary file 5 — Source data Fig. 3 [file 44319_2025_430_MOESM5_ESM.zip › Figure 3/3C/Coomassie CN-PAGE.tiff]

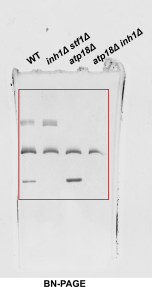

Supplement: Supplementary file 5 — Source data Fig. 3 [file 44319_2025_430_MOESM5_ESM.zip › Figure 3/3C/CV in gel activity BN-PAGE.tiff]

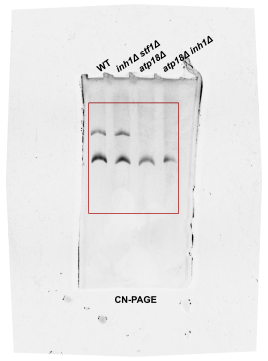

Supplement: Supplementary file 5 — Source data Fig. 3 [file 44319_2025_430_MOESM5_ESM.zip › Figure 3/3C/CV in gel activity CN-PAGE.tiff]

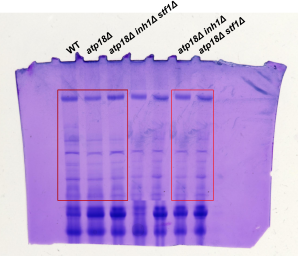

Supplement: Supplementary file 5 — Source data Fig. 3 [file 44319_2025_430_MOESM5_ESM.zip › Figure 3/3D/coomassie.tiff]

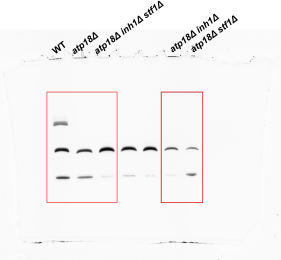

Supplement: Supplementary file 5 — Source data Fig. 3 [file 44319_2025_430_MOESM5_ESM.zip › Figure 3/3D/CV in gel activity.tiff]

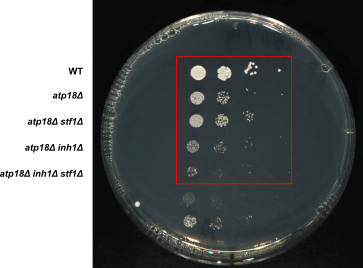

Supplement: Supplementary file 5 — Source data Fig. 3 [file 44319_2025_430_MOESM5_ESM.zip › Figure 3/3G/Drop test.tiff]

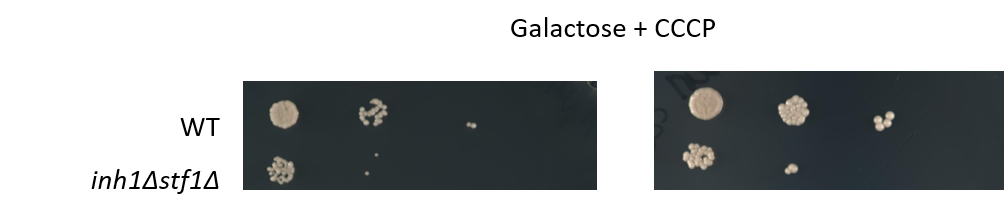

Supplement: Supplementary file 6 — Source data Fig. 4 [file 44319_2025_430_MOESM6_ESM.zip › Figure 4/4A/drop test galactose + CCCP replicate.tif]

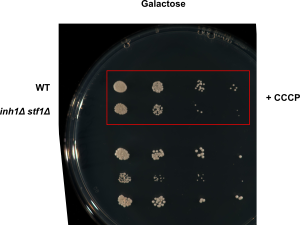

Supplement: Supplementary file 6 — Source data Fig. 4 [file 44319_2025_430_MOESM6_ESM.zip › Figure 4/4A/drop test galactose + CCCP.tiff]

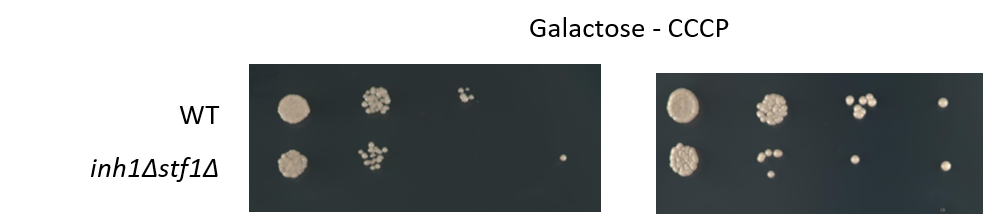

Supplement: Supplementary file 6 — Source data Fig. 4 [file 44319_2025_430_MOESM6_ESM.zip › Figure 4/4A/drop test galactose - CCCP replicate.tif]

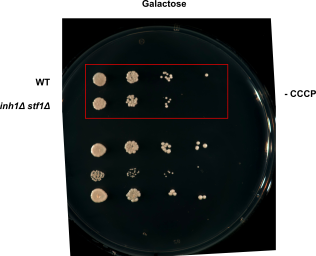

Supplement: Supplementary file 6 — Source data Fig. 4 [file 44319_2025_430_MOESM6_ESM.zip › Figure 4/4A/drop test galactose - CCCP.tiff]

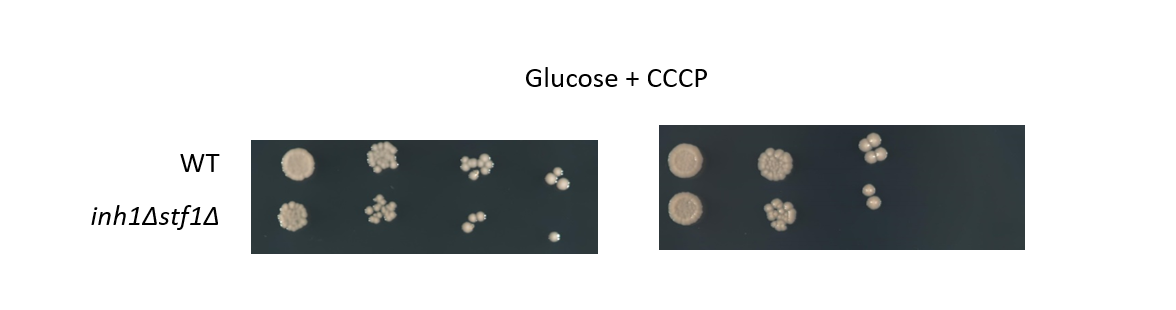

Supplement: Supplementary file 6 — Source data Fig. 4 [file 44319_2025_430_MOESM6_ESM.zip › Figure 4/4A/drop test glucose + CCCP replicate.tif]

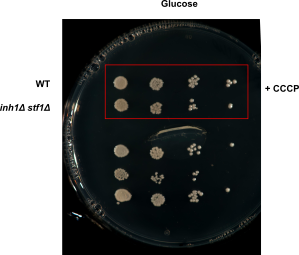

Supplement: Supplementary file 6 — Source data Fig. 4 [file 44319_2025_430_MOESM6_ESM.zip › Figure 4/4A/drop test glucose + CCCP.tiff]

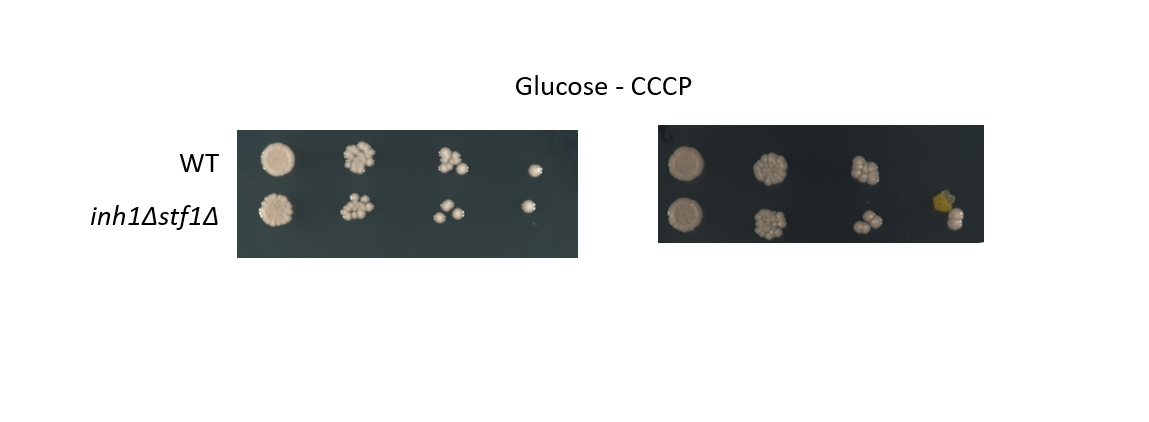

Supplement: Supplementary file 6 — Source data Fig. 4 [file 44319_2025_430_MOESM6_ESM.zip › Figure 4/4A/drop test glucose - CCCP replicate.tif]

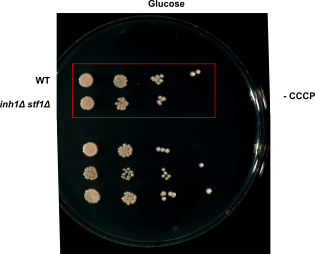

Supplement: Supplementary file 6 — Source data Fig. 4 [file 44319_2025_430_MOESM6_ESM.zip › Figure 4/4A/drop test glucose - CCCP.tiff]

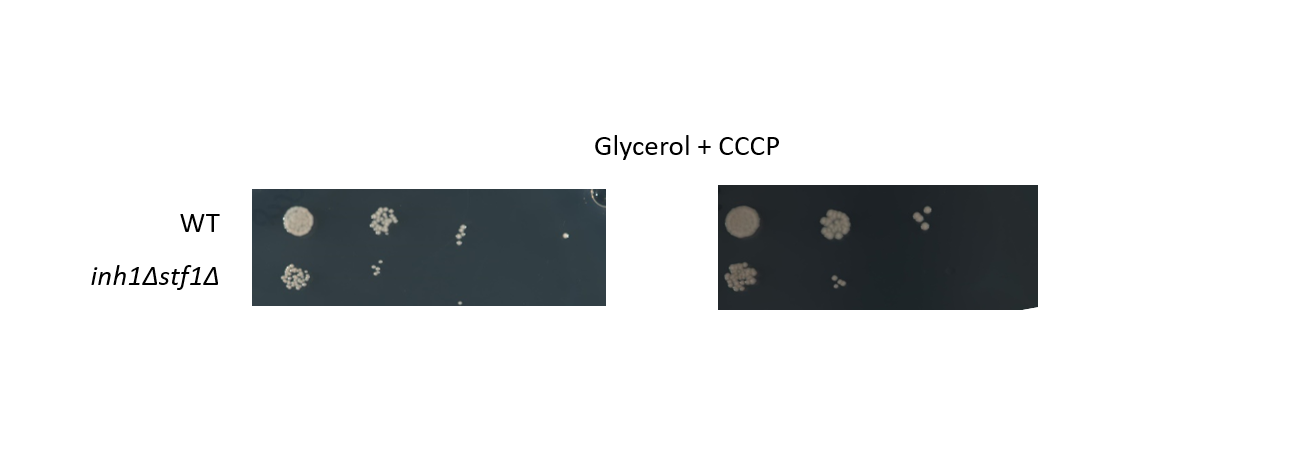

Supplement: Supplementary file 6 — Source data Fig. 4 [file 44319_2025_430_MOESM6_ESM.zip › Figure 4/4A/drop test glycerol + CCCP replicate.tif]

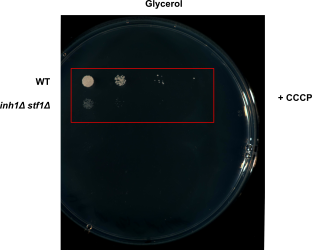

Supplement: Supplementary file 6 — Source data Fig. 4 [file 44319_2025_430_MOESM6_ESM.zip › Figure 4/4A/drop test glycerol + CCCP.tiff]

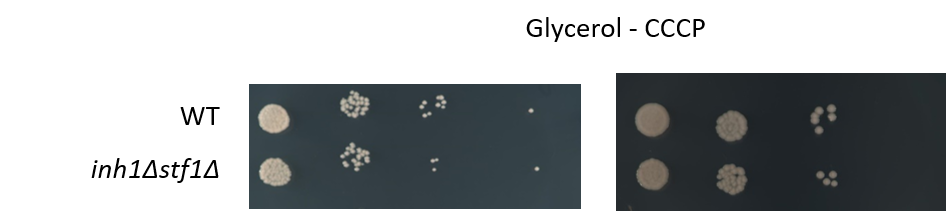

Supplement: Supplementary file 6 — Source data Fig. 4 [file 44319_2025_430_MOESM6_ESM.zip › Figure 4/4A/drop test glycerol - CCCP replicate.tif]

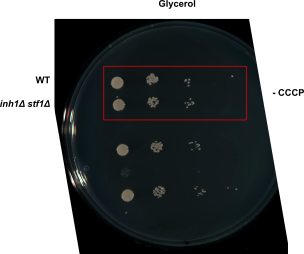

Supplement: Supplementary file 6 — Source data Fig. 4 [file 44319_2025_430_MOESM6_ESM.zip › Figure 4/4A/drop test glycerol - CCCP.tiff]

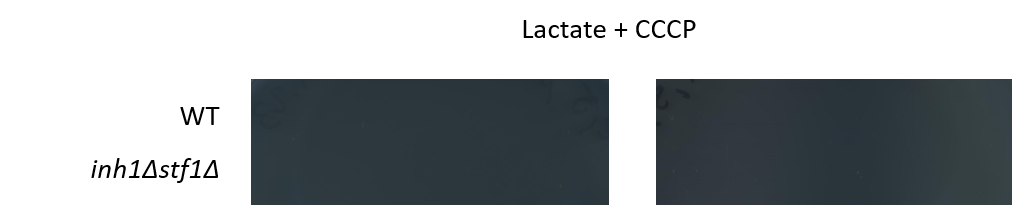

Supplement: Supplementary file 6 — Source data Fig. 4 [file 44319_2025_430_MOESM6_ESM.zip › Figure 4/4A/drop test lactate + CCCP replicate.tif]

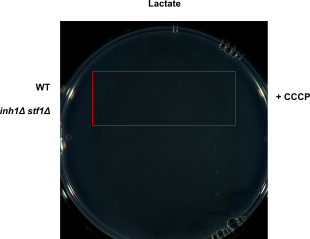

Supplement: Supplementary file 6 — Source data Fig. 4 [file 44319_2025_430_MOESM6_ESM.zip › Figure 4/4A/drop test lactate + CCCP.tiff]

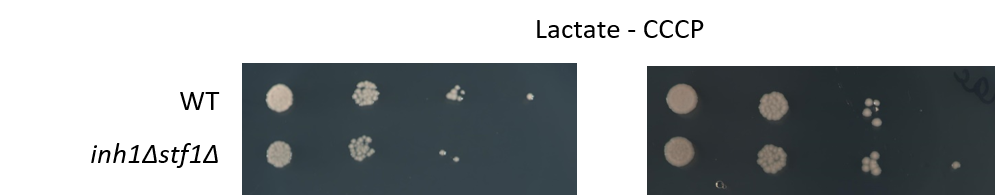

Supplement: Supplementary file 6 — Source data Fig. 4 [file 44319_2025_430_MOESM6_ESM.zip › Figure 4/4A/drop test lactate - CCCP replicate.tif]

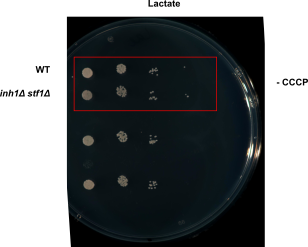

Supplement: Supplementary file 6 — Source data Fig. 4 [file 44319_2025_430_MOESM6_ESM.zip › Figure 4/4A/drop test lactate - CCCP.tiff]

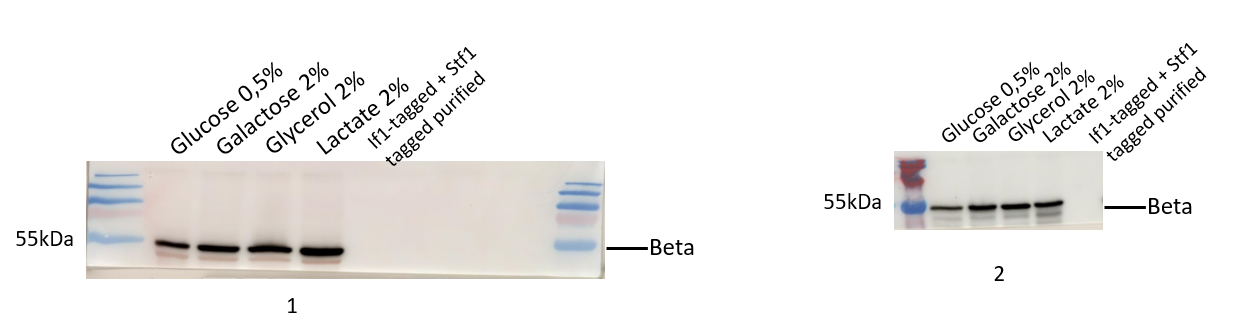

Supplement: Supplementary file 7 — Source data Fig. 5 [file 44319_2025_430_MOESM7_ESM.zip › Figure 5/5A/western beta replicate.tif]

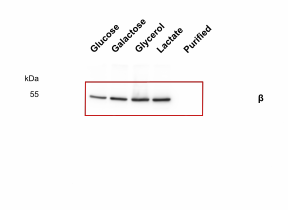

Supplement: Supplementary file 7 — Source data Fig. 5 [file 44319_2025_430_MOESM7_ESM.zip › Figure 5/5A/western beta.tiff]

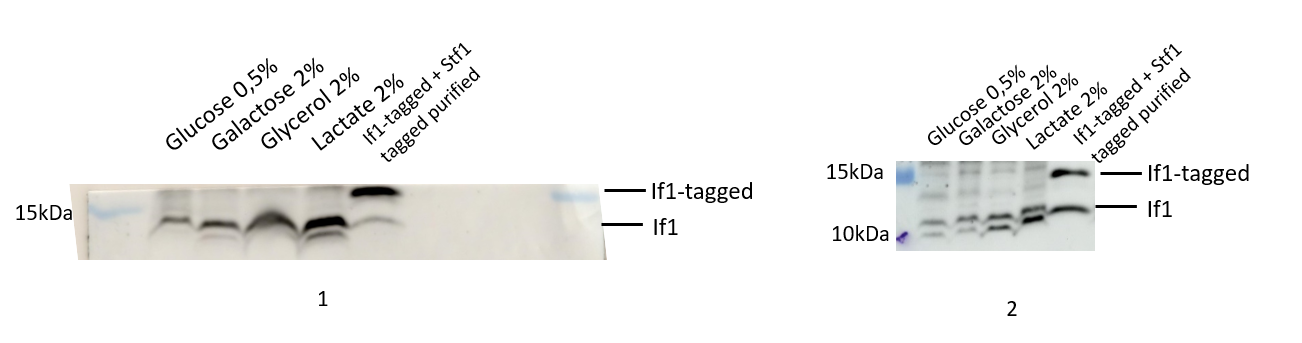

Supplement: Supplementary file 7 — Source data Fig. 5 [file 44319_2025_430_MOESM7_ESM.zip › Figure 5/5A/western If1 replicate.tif]

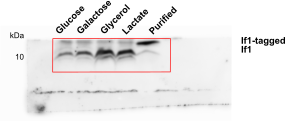

Supplement: Supplementary file 7 — Source data Fig. 5 [file 44319_2025_430_MOESM7_ESM.zip › Figure 5/5A/western if1.tiff]

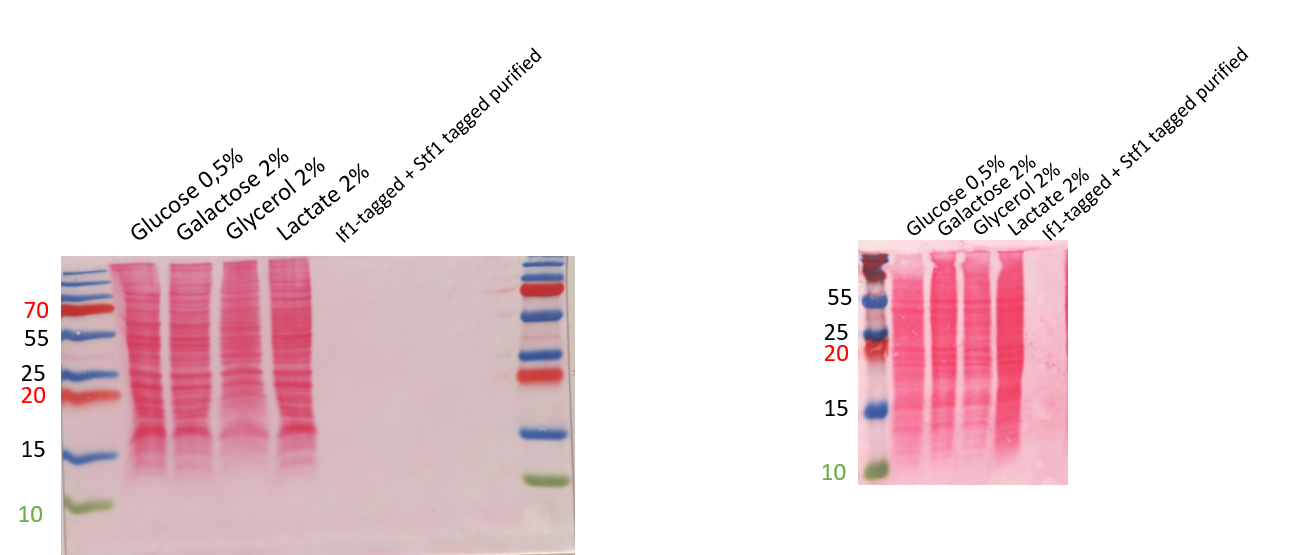

Supplement: Supplementary file 7 — Source data Fig. 5 [file 44319_2025_430_MOESM7_ESM.zip › Figure 5/5A/western ponceau replicate.tif]

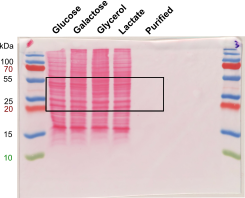

Supplement: Supplementary file 7 — Source data Fig. 5 [file 44319_2025_430_MOESM7_ESM.zip › Figure 5/5A/western ponceau.tiff]

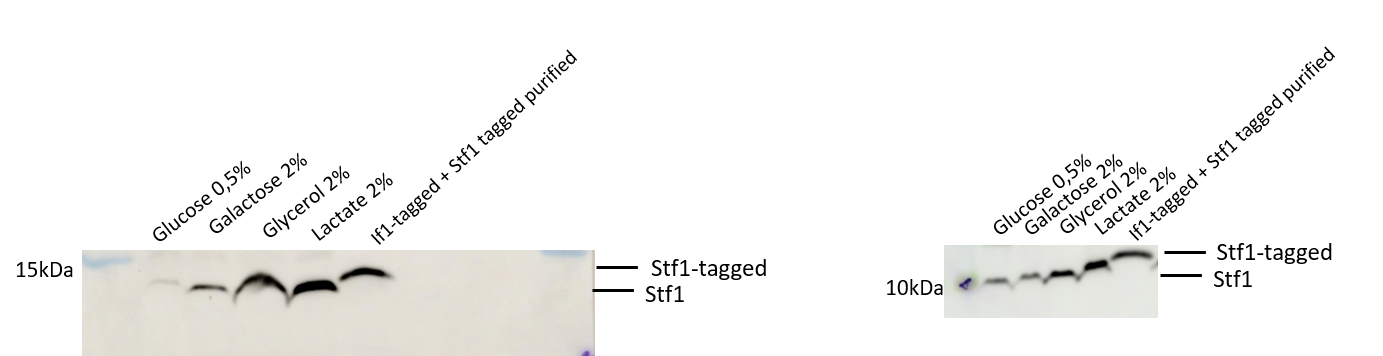

Supplement: Supplementary file 7 — Source data Fig. 5 [file 44319_2025_430_MOESM7_ESM.zip › Figure 5/5A/western Stf1 replicate.tif]

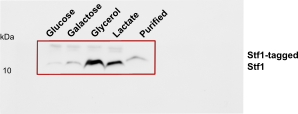

Supplement: Supplementary file 7 — Source data Fig. 5 [file 44319_2025_430_MOESM7_ESM.zip › Figure 5/5A/western stf1.tiff]

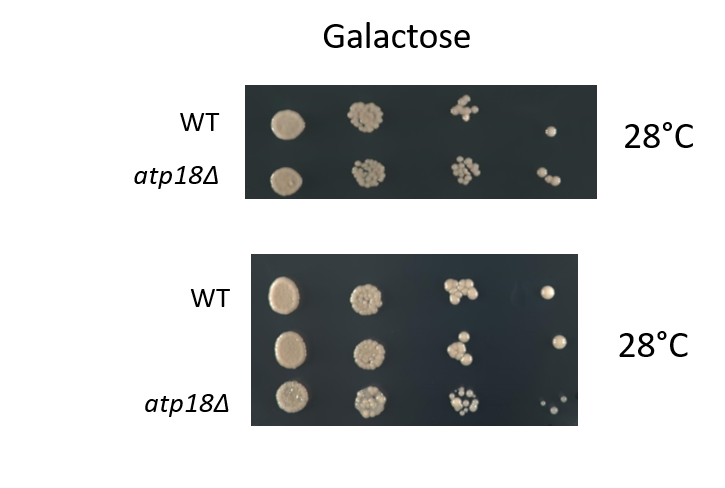

Supplement: Supplementary file 7 — Source data Fig. 5 [file 44319_2025_430_MOESM7_ESM.zip › Figure 5/5C/drop test galactose 28°C replicate.tif]

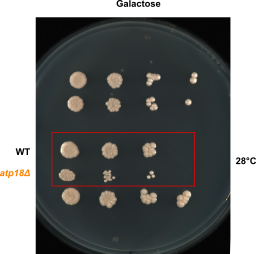

Supplement: Supplementary file 7 — Source data Fig. 5 [file 44319_2025_430_MOESM7_ESM.zip › Figure 5/5C/drop test galactose 28°C.tiff]

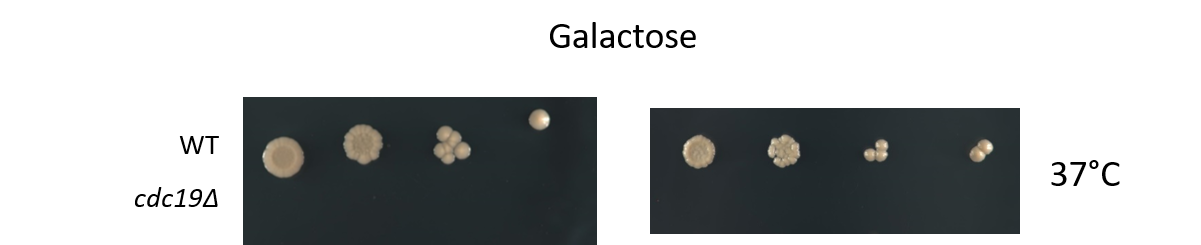

Supplement: Supplementary file 7 — Source data Fig. 5 [file 44319_2025_430_MOESM7_ESM.zip › Figure 5/5C/drop test galactose 37°C replicate.tif]

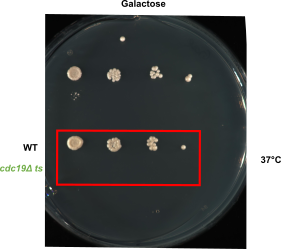

Supplement: Supplementary file 7 — Source data Fig. 5 [file 44319_2025_430_MOESM7_ESM.zip › Figure 5/5C/drop test galactose 37°C.tiff]

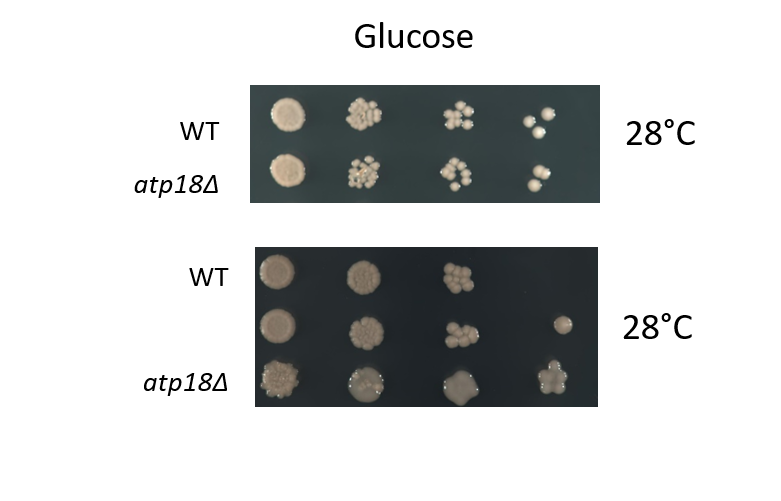

Supplement: Supplementary file 7 — Source data Fig. 5 [file 44319_2025_430_MOESM7_ESM.zip › Figure 5/5C/drop test glucose 28°C replicate.tif]

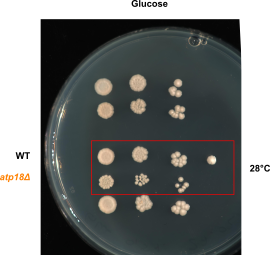

Supplement: Supplementary file 7 — Source data Fig. 5 [file 44319_2025_430_MOESM7_ESM.zip › Figure 5/5C/drop test glucose 28°C.tiff]

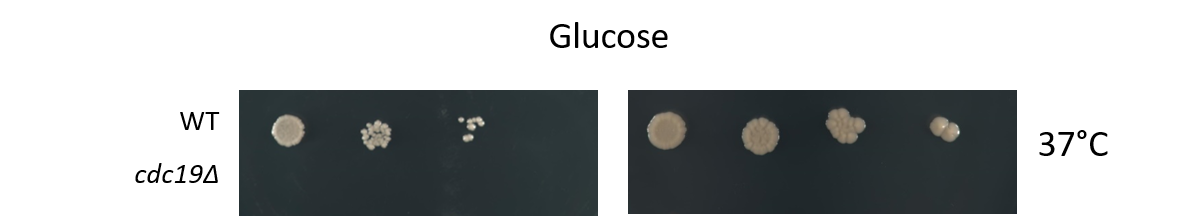

Supplement: Supplementary file 7 — Source data Fig. 5 [file 44319_2025_430_MOESM7_ESM.zip › Figure 5/5C/drop test glucose 37°C replicate.tif]

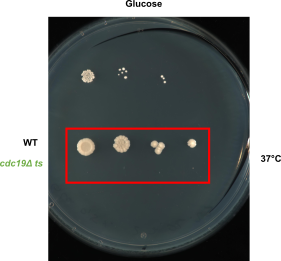

Supplement: Supplementary file 7 — Source data Fig. 5 [file 44319_2025_430_MOESM7_ESM.zip › Figure 5/5C/drop test glucose 37°C.tiff]

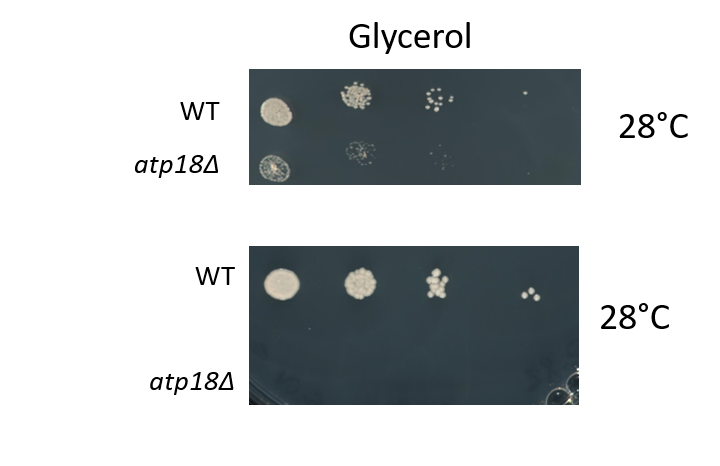

Supplement: Supplementary file 7 — Source data Fig. 5 [file 44319_2025_430_MOESM7_ESM.zip › Figure 5/5C/drop test glycerol 28°C replicate.tif]

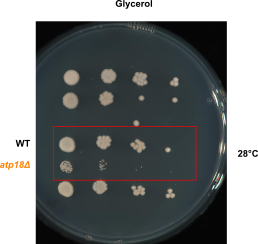

Supplement: Supplementary file 7 — Source data Fig. 5 [file 44319_2025_430_MOESM7_ESM.zip › Figure 5/5C/drop test glycerol 28°C.tiff]

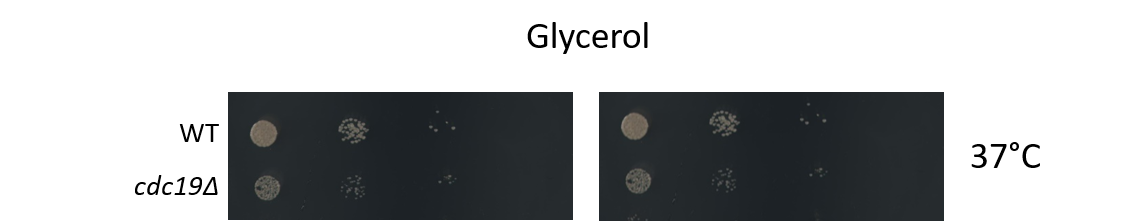

Supplement: Supplementary file 7 — Source data Fig. 5 [file 44319_2025_430_MOESM7_ESM.zip › Figure 5/5C/drop test glycerol 37°C replicate.tif]

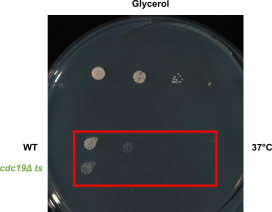

Supplement: Supplementary file 7 — Source data Fig. 5 [file 44319_2025_430_MOESM7_ESM.zip › Figure 5/5C/drop test glycerol 37°C.tiff]

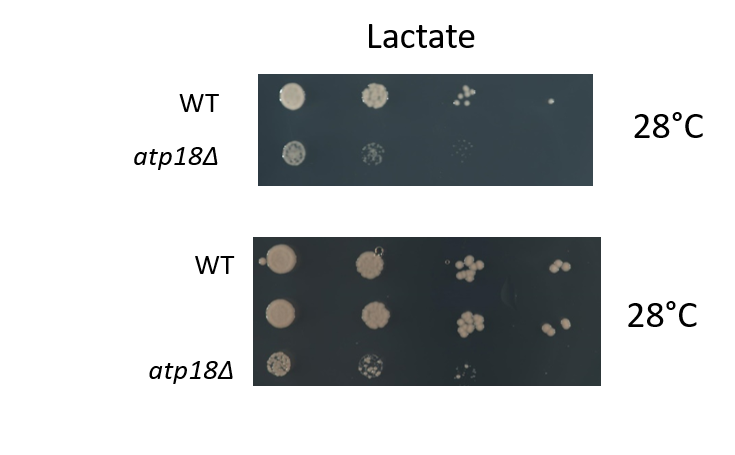

Supplement: Supplementary file 7 — Source data Fig. 5 [file 44319_2025_430_MOESM7_ESM.zip › Figure 5/5C/drop test lactate 28°C replicate.tif]

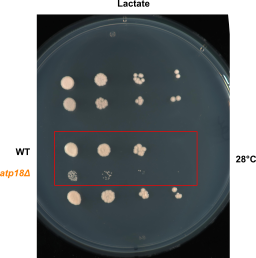

Supplement: Supplementary file 7 — Source data Fig. 5 [file 44319_2025_430_MOESM7_ESM.zip › Figure 5/5C/drop test lactate 28°C.tiff]

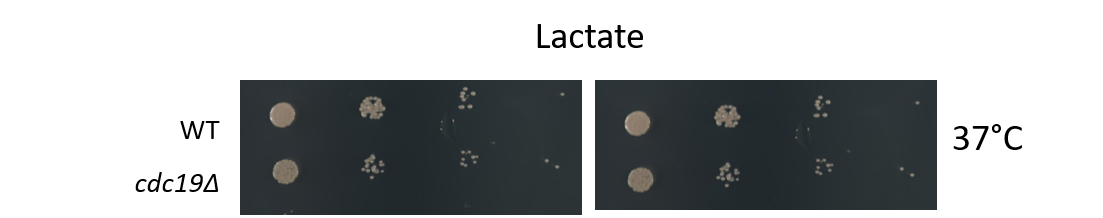

Supplement: Supplementary file 7 — Source data Fig. 5 [file 44319_2025_430_MOESM7_ESM.zip › Figure 5/5C/drop test lactate 37°C replicate.tif]

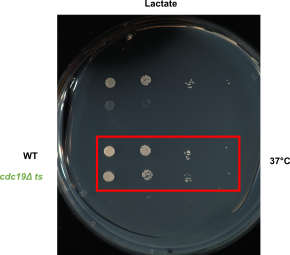

Supplement: Supplementary file 7 — Source data Fig. 5 [file 44319_2025_430_MOESM7_ESM.zip › Figure 5/5C/drop test lactate 37°C.tiff]

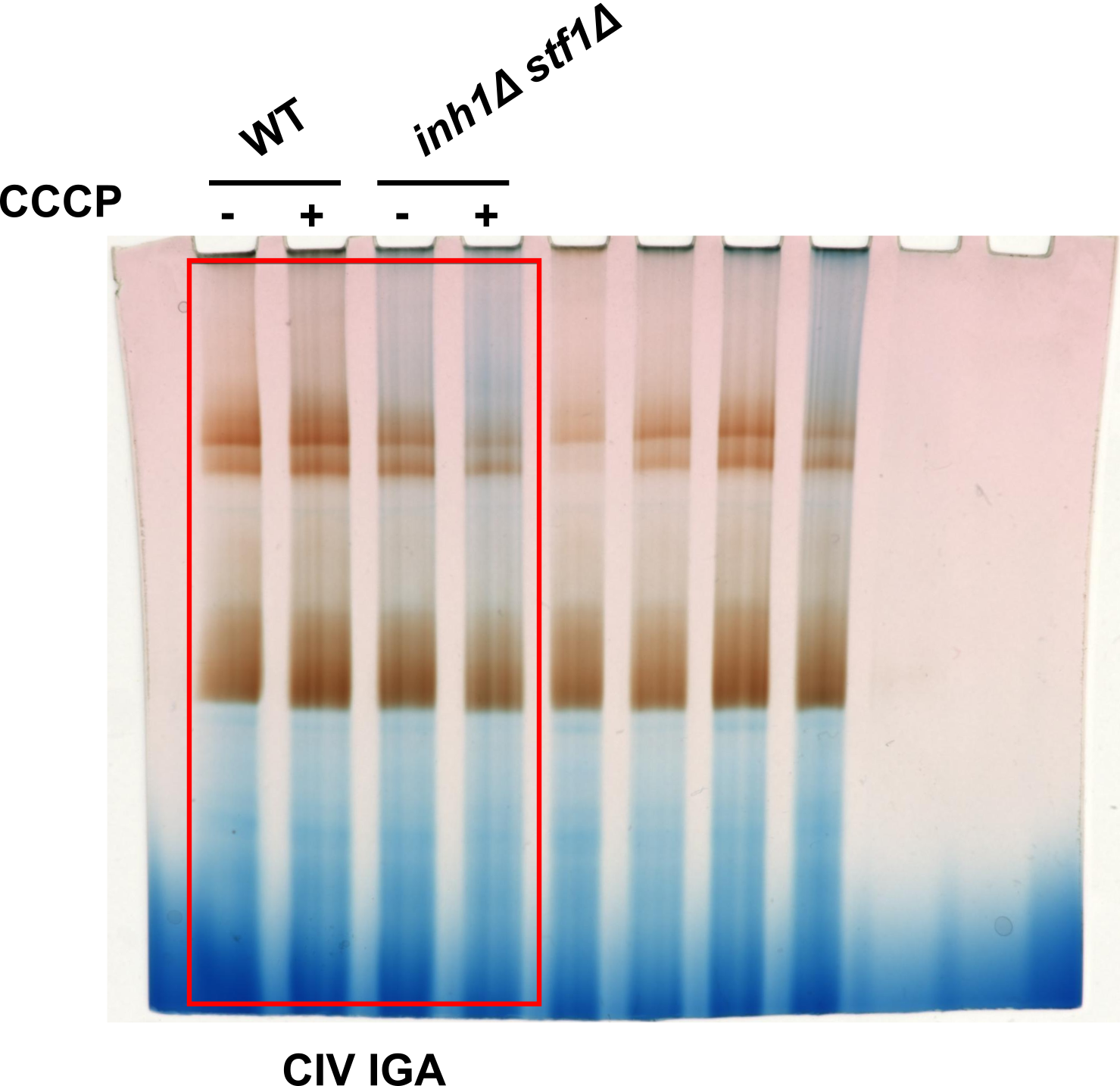

Supplement: Supplementary file 7 — Source data Fig. 5 [file 44319_2025_430_MOESM7_ESM.zip › Figure 5/5F/CIV in gel activity.tiff]

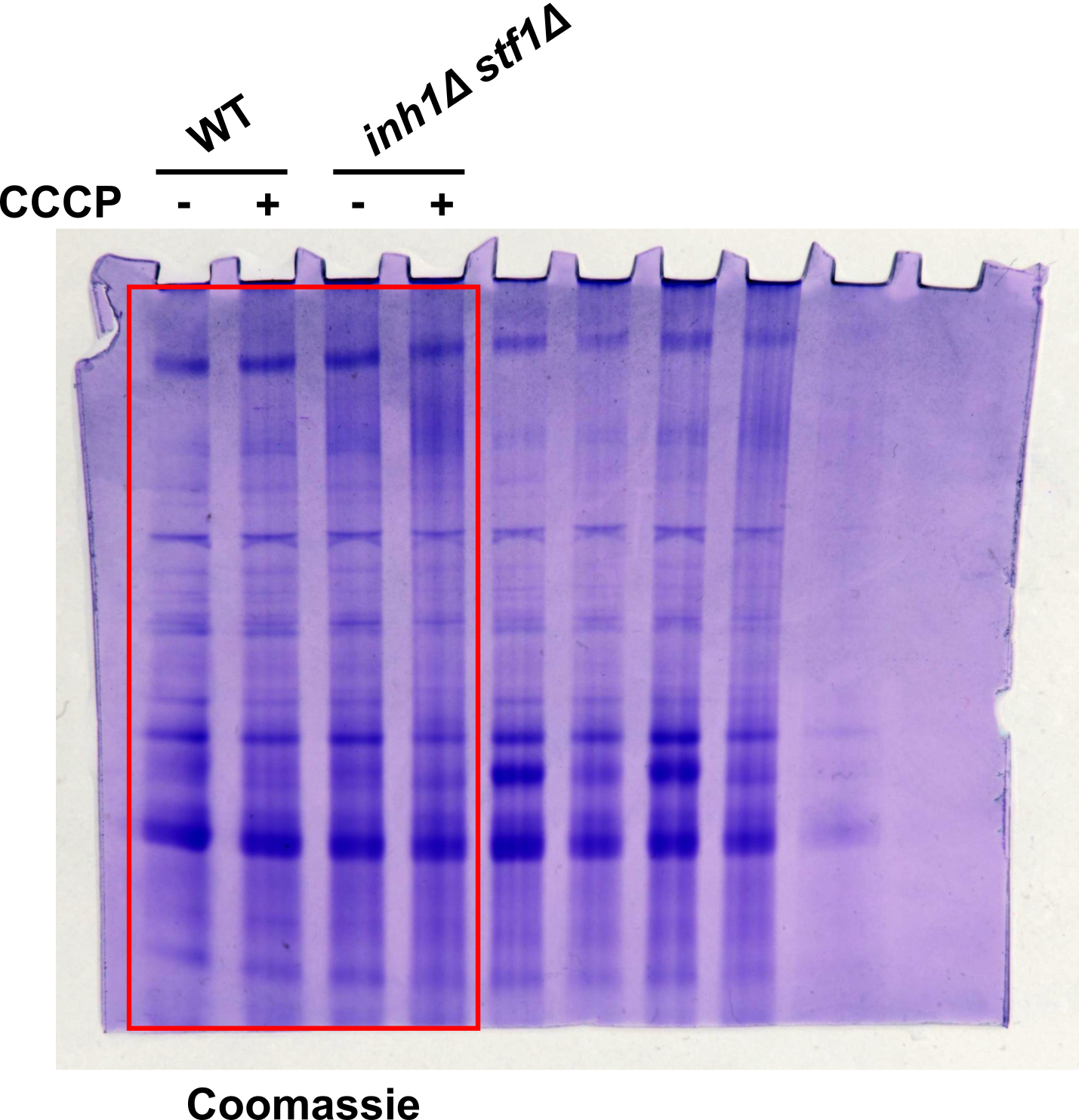

Supplement: Supplementary file 7 — Source data Fig. 5 [file 44319_2025_430_MOESM7_ESM.zip › Figure 5/5F/Coomassie.tiff]

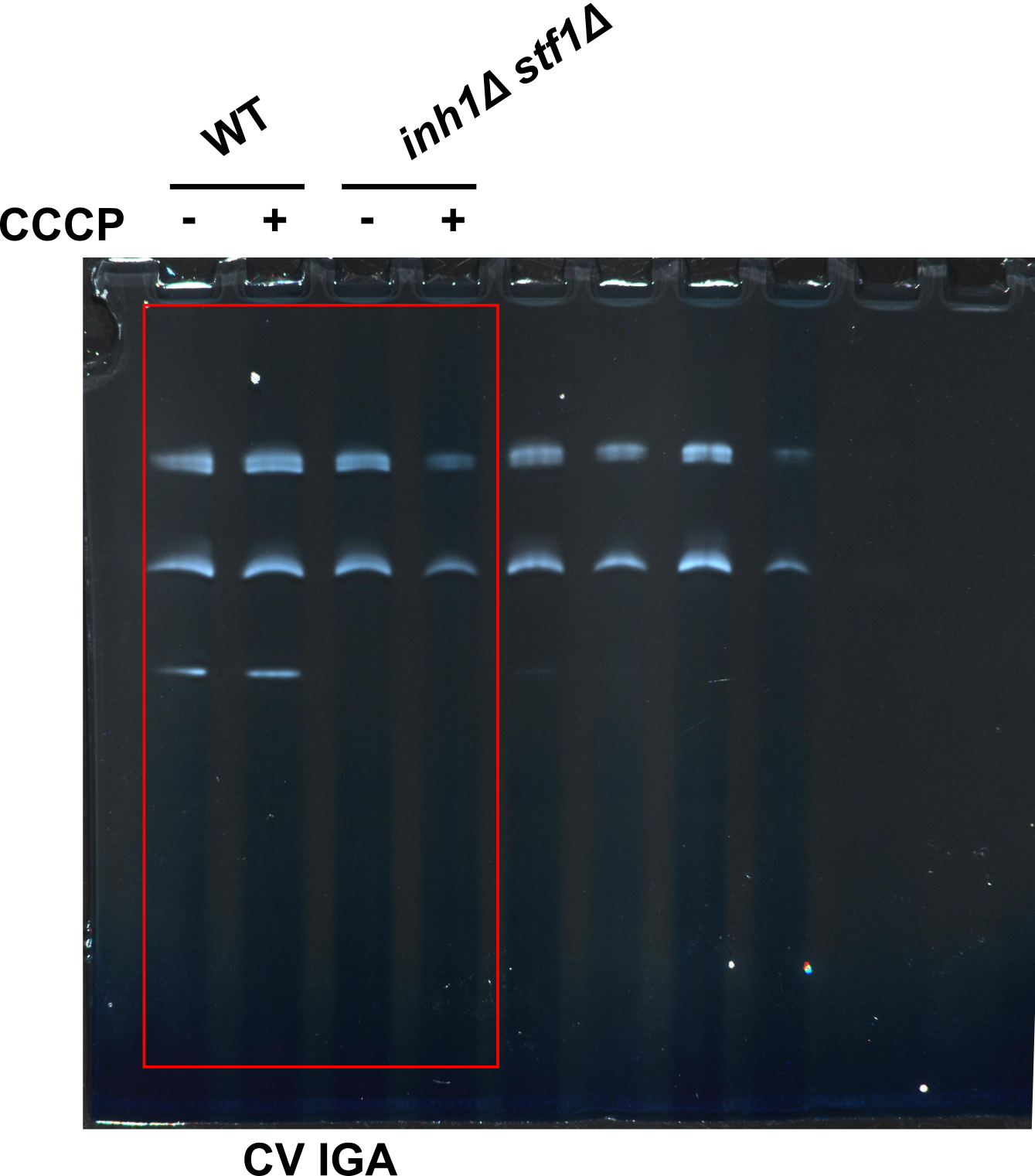

Supplement: Supplementary file 7 — Source data Fig. 5 [file 44319_2025_430_MOESM7_ESM.zip › Figure 5/5F/CV in gel activity.tiff]

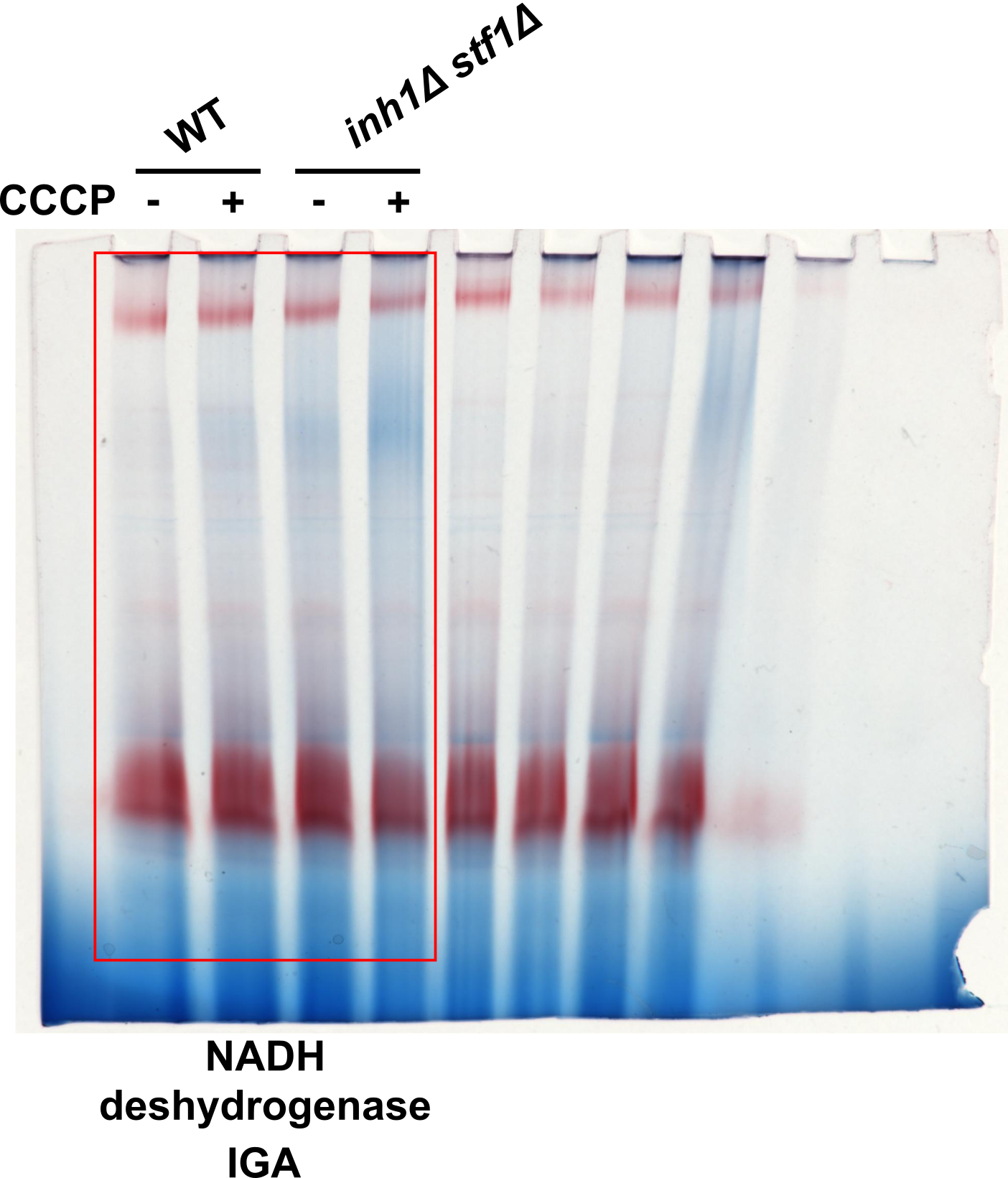

Supplement: Supplementary file 7 — Source data Fig. 5 [file 44319_2025_430_MOESM7_ESM.zip › Figure 5/5F/NADH deshydrogenase in gel activity.tiff]

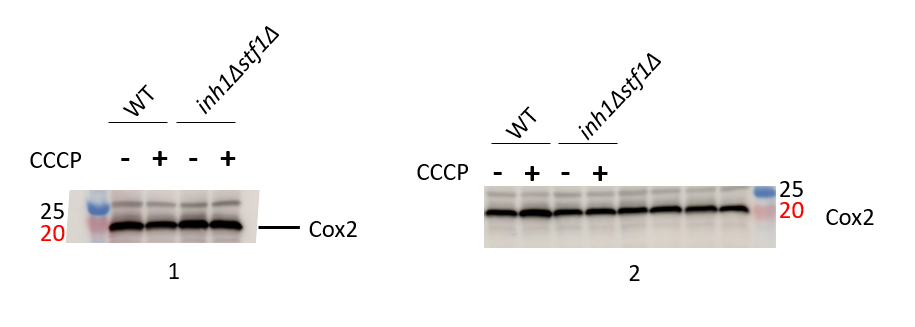

Supplement: Supplementary file 7 — Source data Fig. 5 [file 44319_2025_430_MOESM7_ESM.zip › Figure 5/5I/western cox2 replicate.tif]

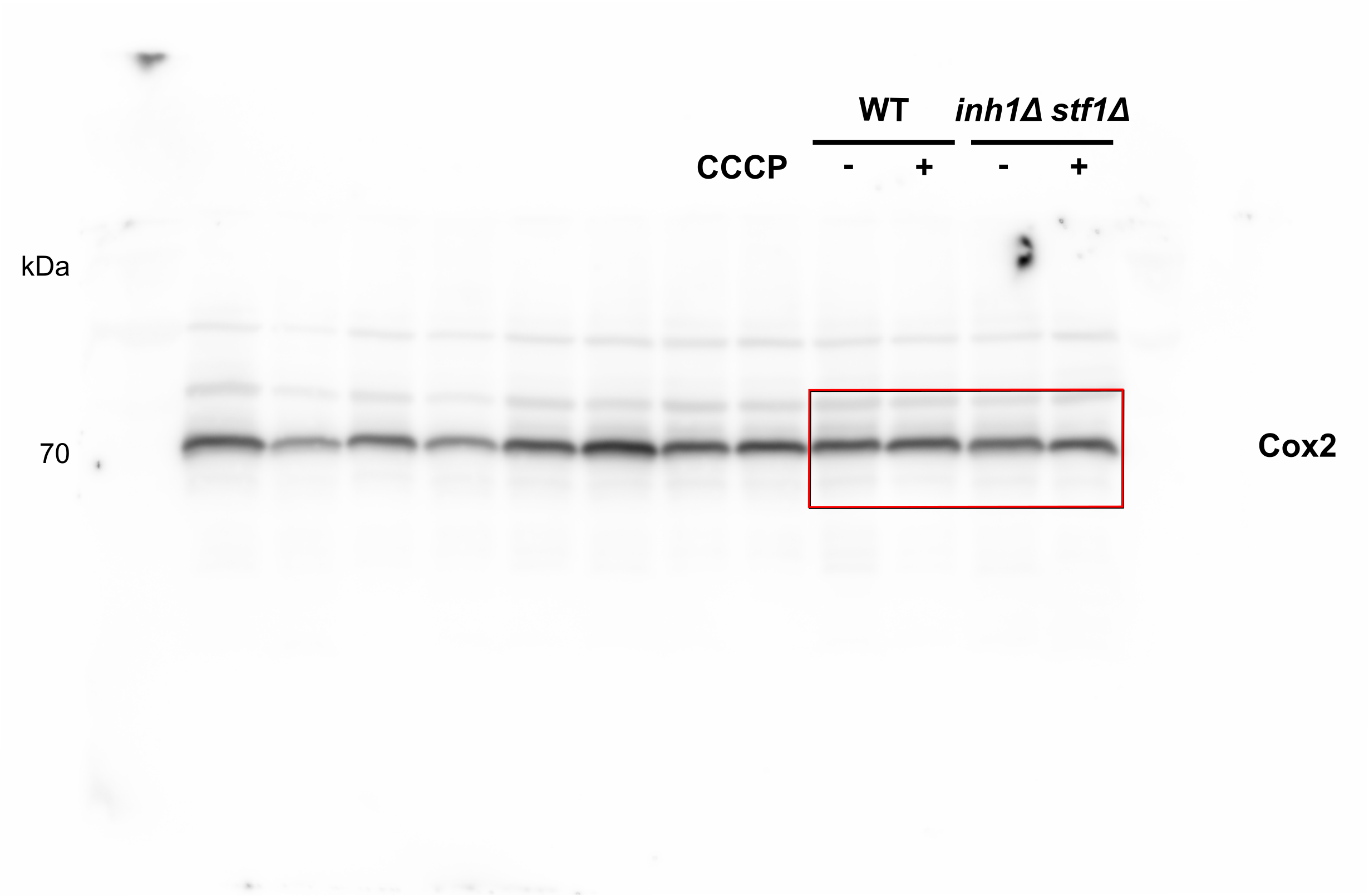

Supplement: Supplementary file 7 — Source data Fig. 5 [file 44319_2025_430_MOESM7_ESM.zip › Figure 5/5I/western cox2.tiff]

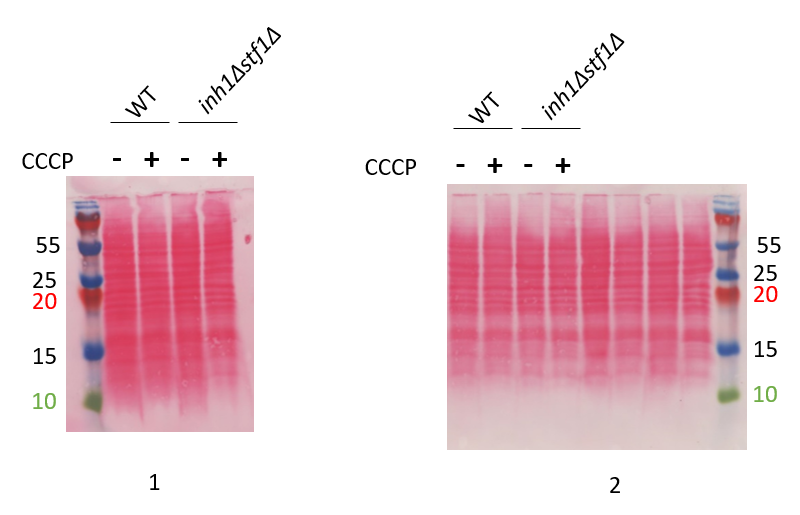

Supplement: Supplementary file 7 — Source data Fig. 5 [file 44319_2025_430_MOESM7_ESM.zip › Figure 5/5I/western ponceau replicate.tif]

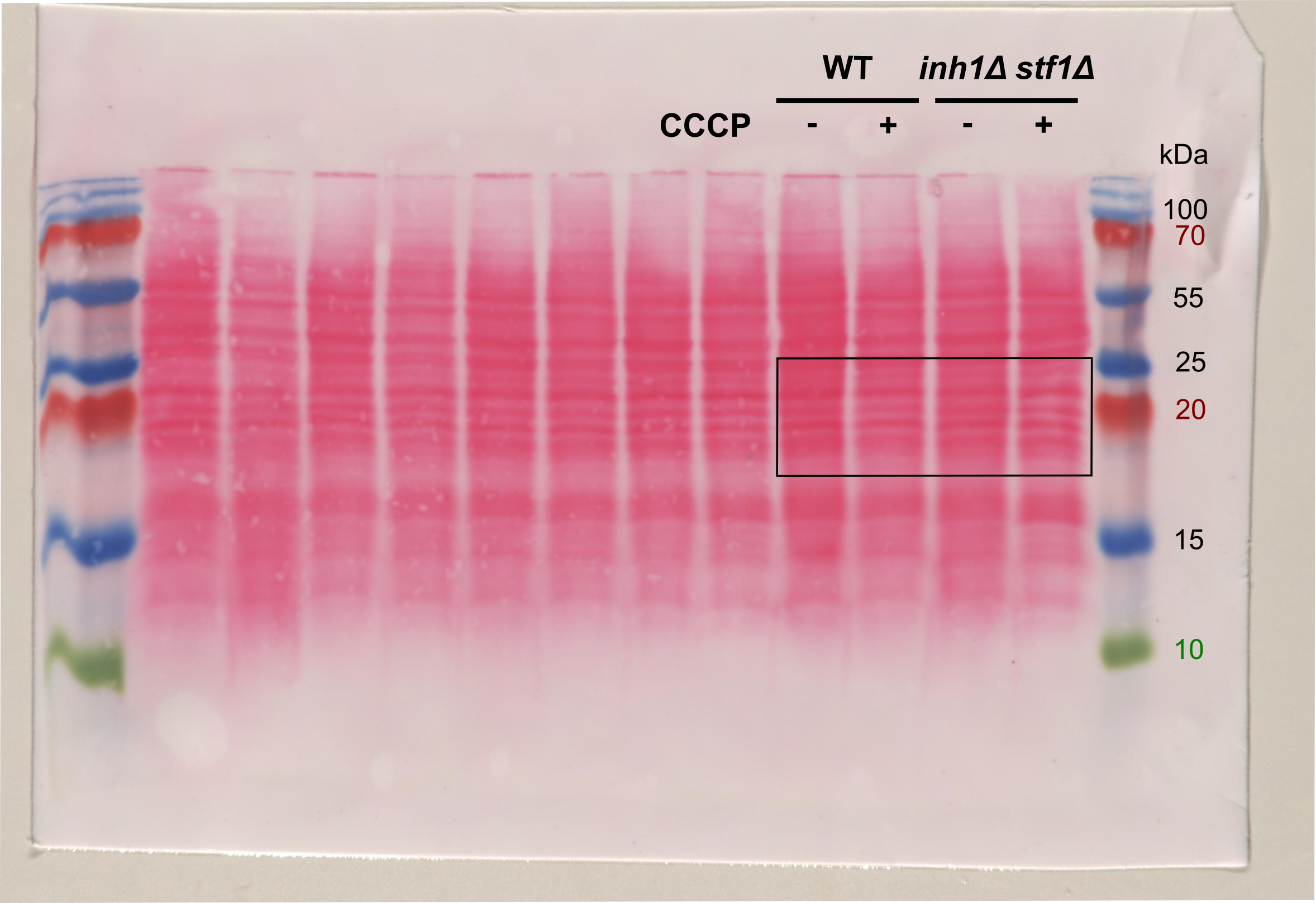

Supplement: Supplementary file 7 — Source data Fig. 5 [file 44319_2025_430_MOESM7_ESM.zip › Figure 5/5I/western ponceau.tiff]

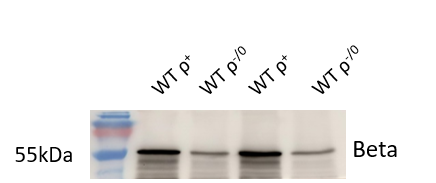

Supplement: Supplementary file 8 — Source data Fig. 6 [file 44319_2025_430_MOESM8_ESM.zip › Figure 6/6B/western beta replicate.tif]
