# Supplementary figures and images for: An alternative mechanism by which If1 prevents ATP hydrolysis by the ATP synthase subcomplex in S. cerevisiae (part 2 of 2)
Source: EMBO Rep. 2025 Jun 9;26(13):3305–26. doi: 10.1038/s44319-025-00430-8 (PMC12238618; doi:10.1038/s44319-025-00430-8)

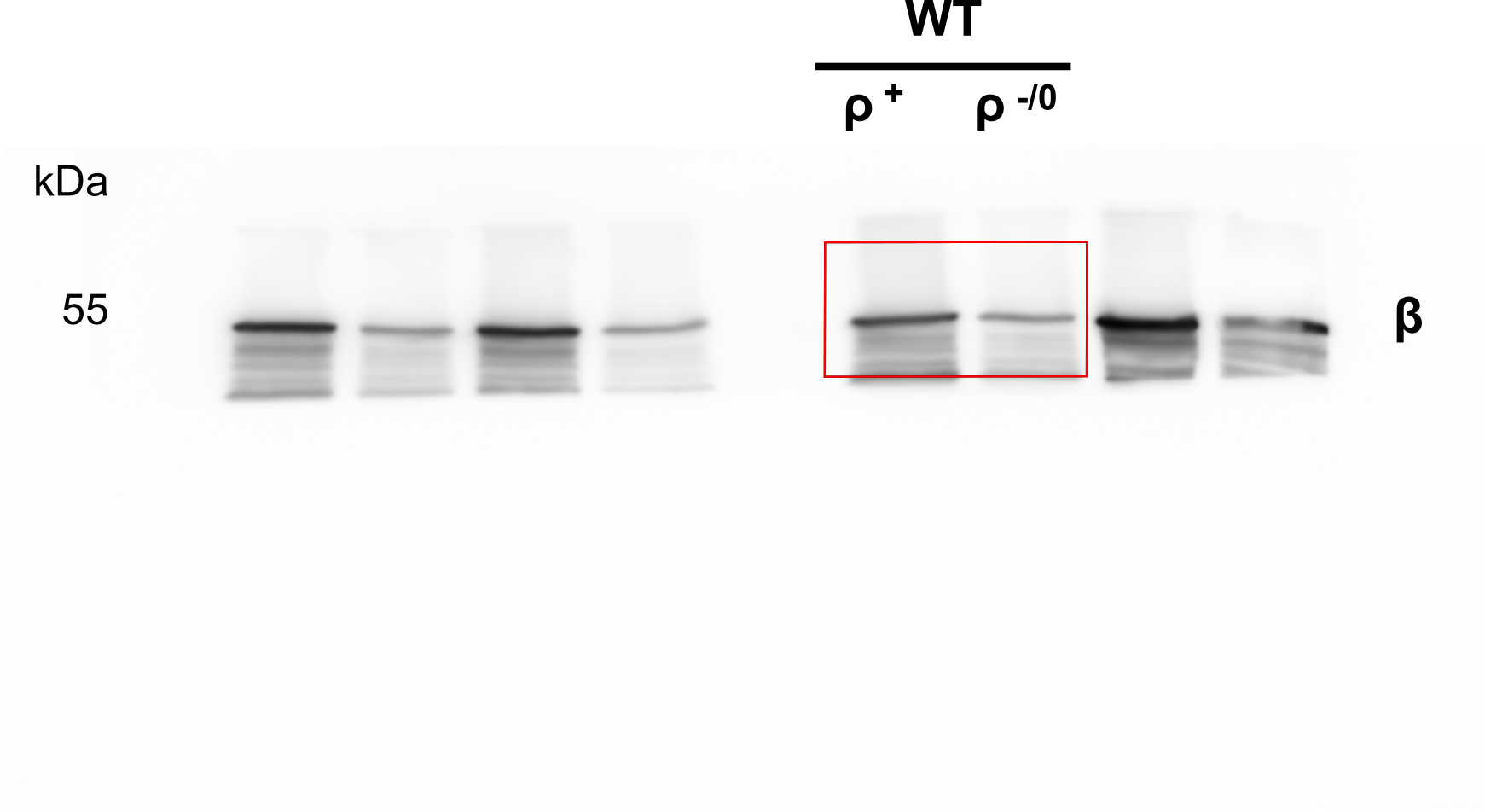

Supplement: Supplementary file 8 — Source data Fig. 6 [file 44319_2025_430_MOESM8_ESM.zip › Figure 6/6B/western beta.tiff]

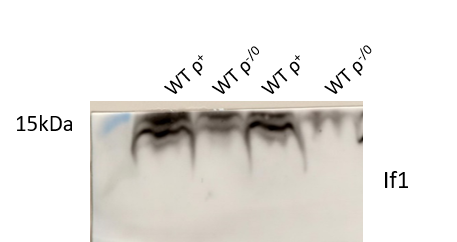

Supplement: Supplementary file 8 — Source data Fig. 6 [file 44319_2025_430_MOESM8_ESM.zip › Figure 6/6B/western If1 replicate.tif]

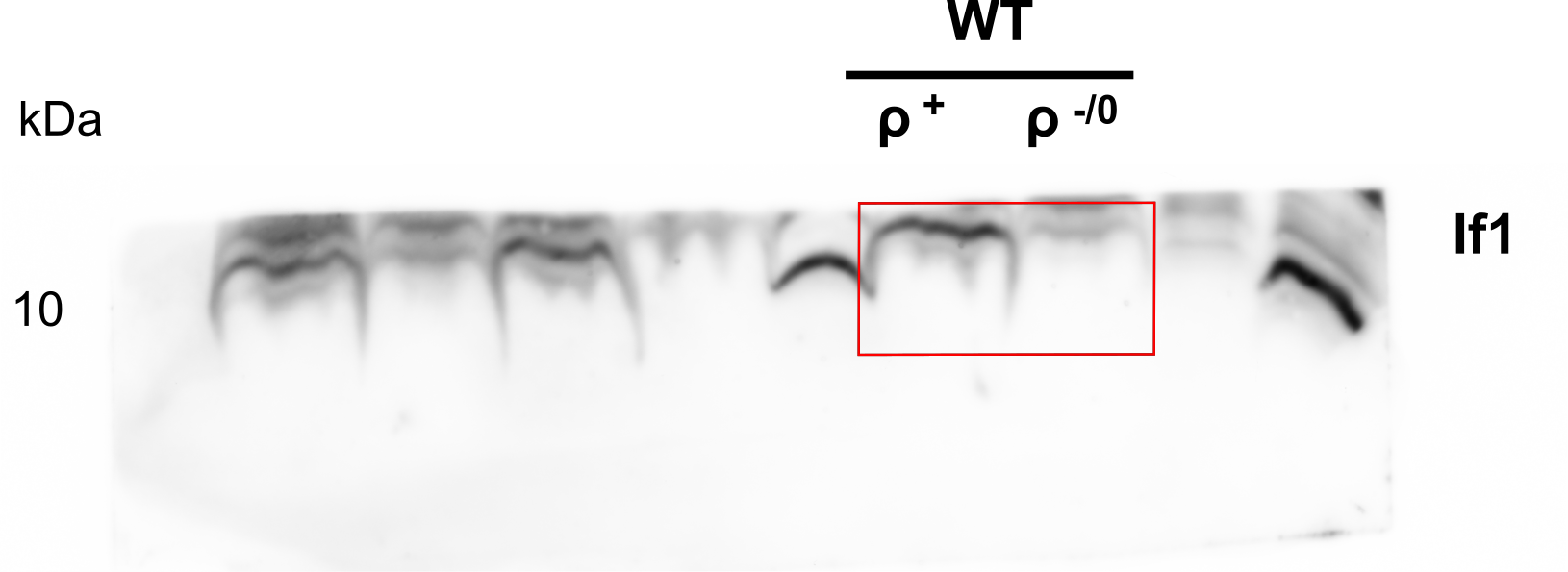

Supplement: Supplementary file 8 — Source data Fig. 6 [file 44319_2025_430_MOESM8_ESM.zip › Figure 6/6B/western If1.tiff]

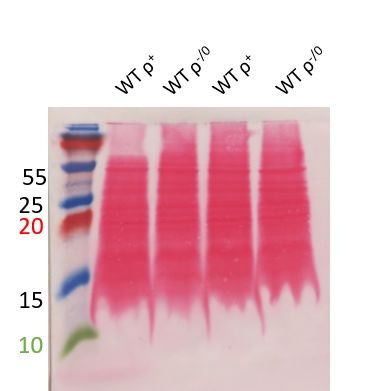

Supplement: Supplementary file 8 — Source data Fig. 6 [file 44319_2025_430_MOESM8_ESM.zip › Figure 6/6B/western ponceau replicate.tif]

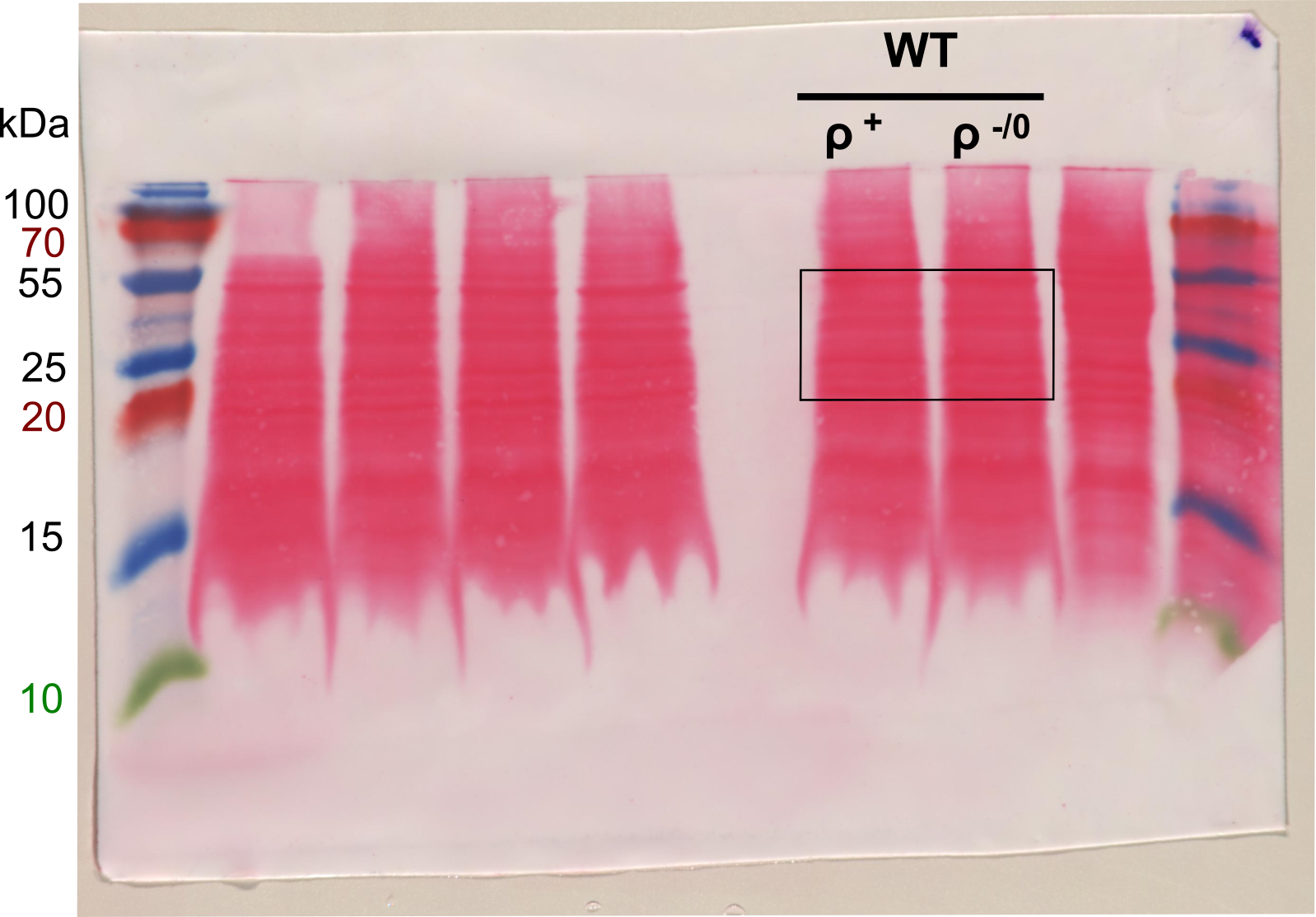

Supplement: Supplementary file 8 — Source data Fig. 6 [file 44319_2025_430_MOESM8_ESM.zip › Figure 6/6B/western ponceau.tiff]

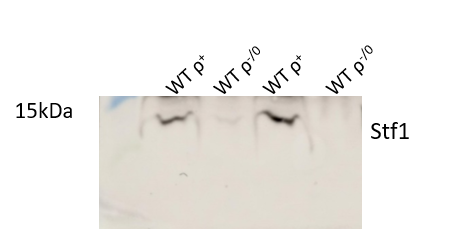

Supplement: Supplementary file 8 — Source data Fig. 6 [file 44319_2025_430_MOESM8_ESM.zip › Figure 6/6B/western Stf1 replicate.tif]

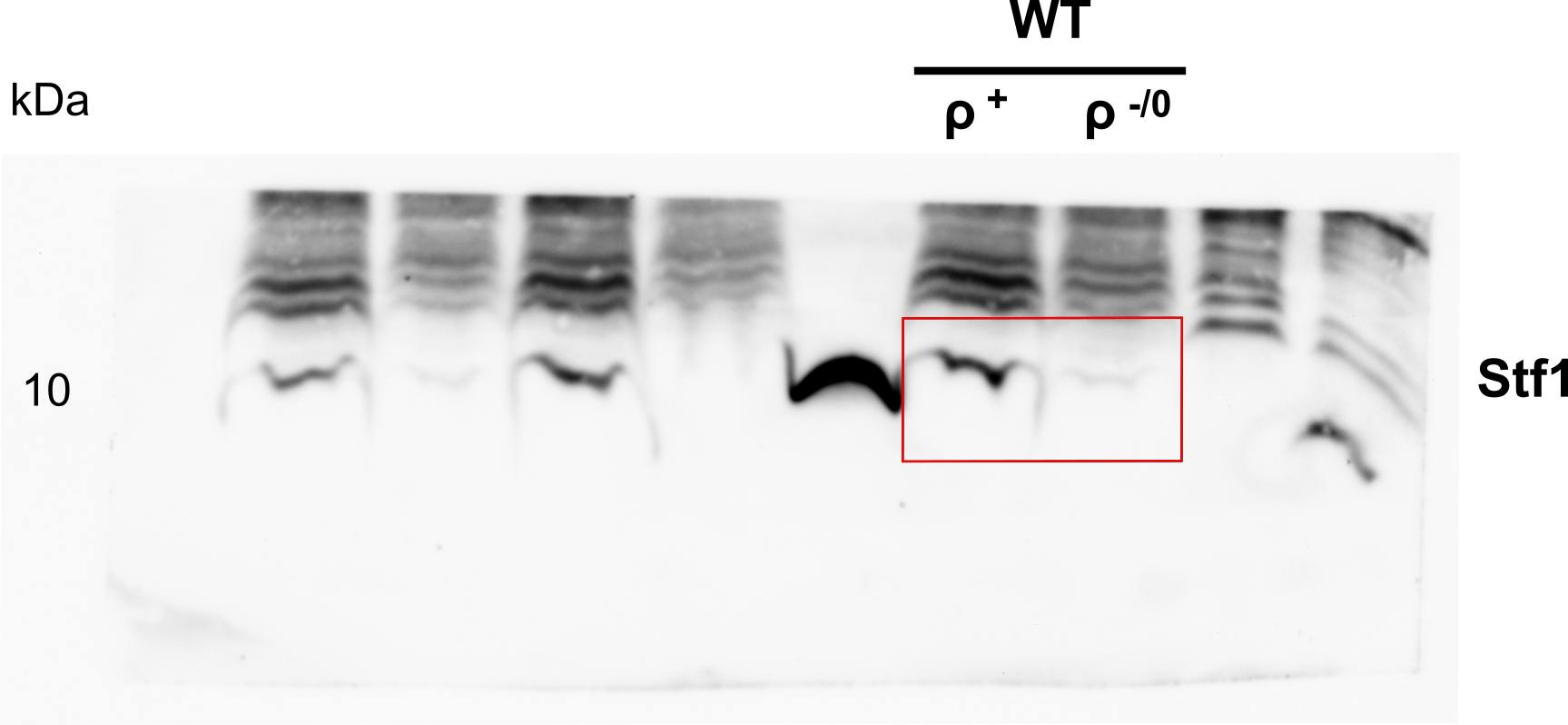

Supplement: Supplementary file 8 — Source data Fig. 6 [file 44319_2025_430_MOESM8_ESM.zip › Figure 6/6B/western Stf1.tiff]

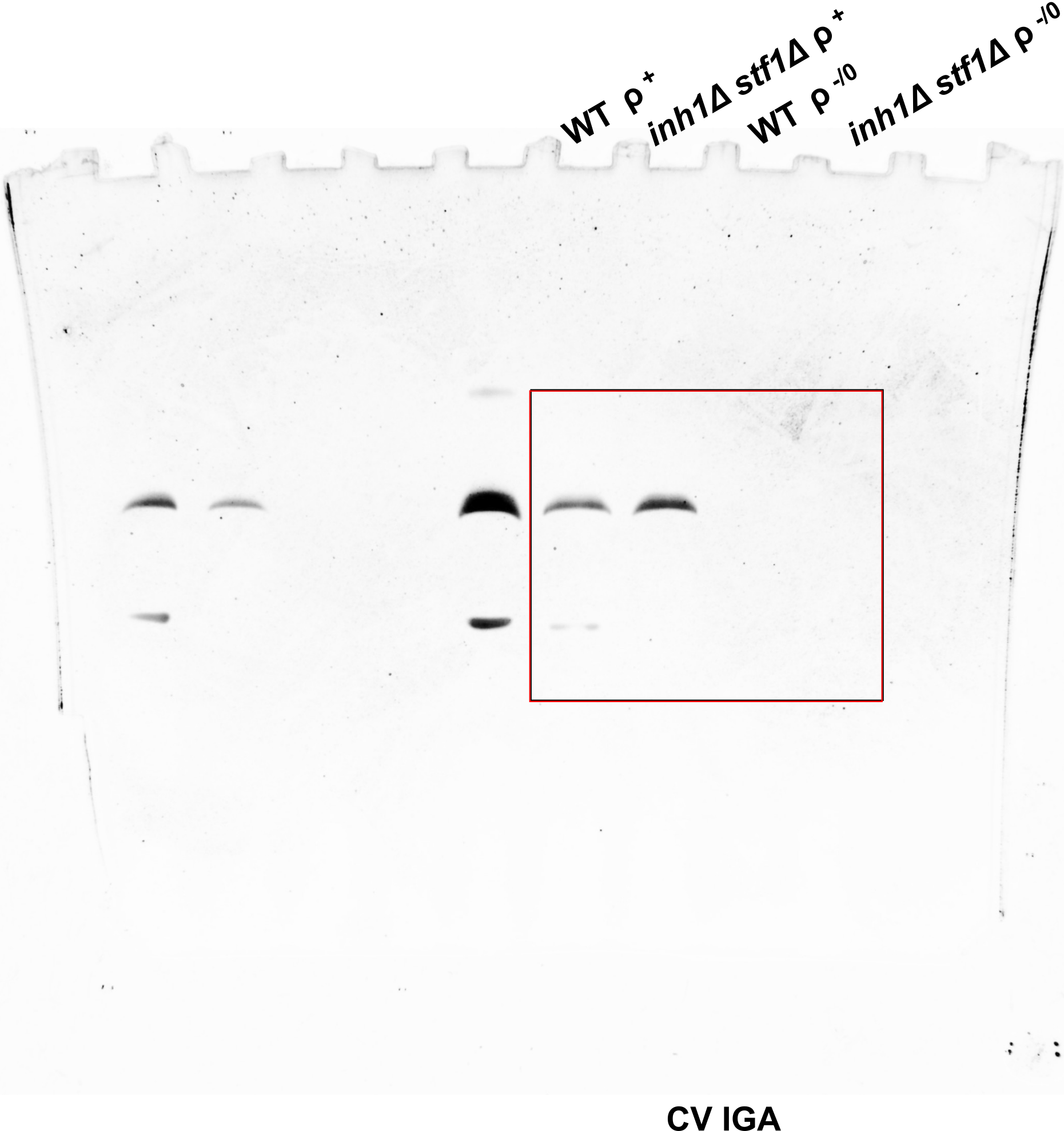

Supplement: Supplementary file 8 — Source data Fig. 6 [file 44319_2025_430_MOESM8_ESM.zip › Figure 6/6E/CV in gel activity.tiff]

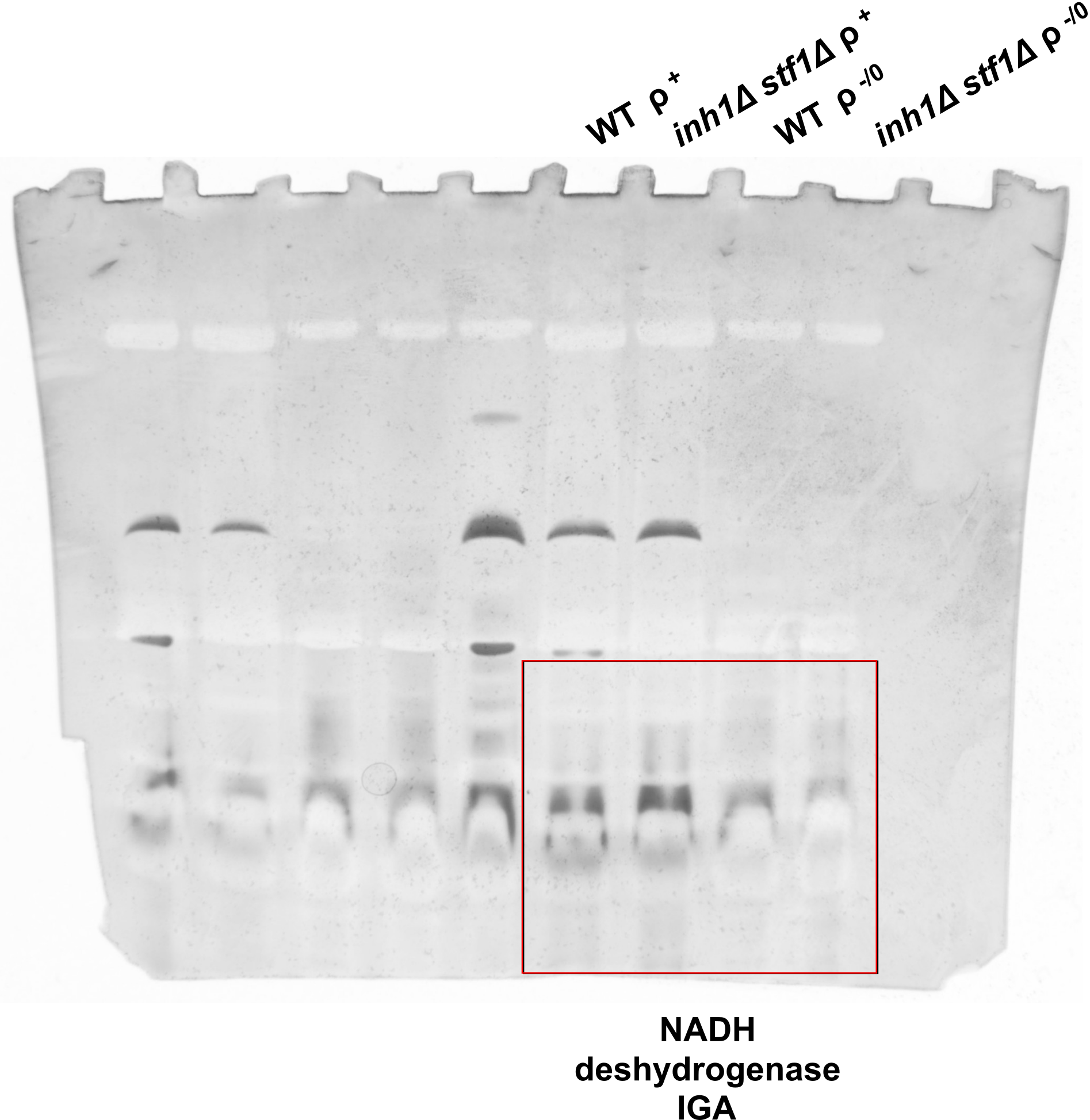

Supplement: Supplementary file 8 — Source data Fig. 6 [file 44319_2025_430_MOESM8_ESM.zip › Figure 6/6E/NADH deshydrogenase in gel activity.tiff]

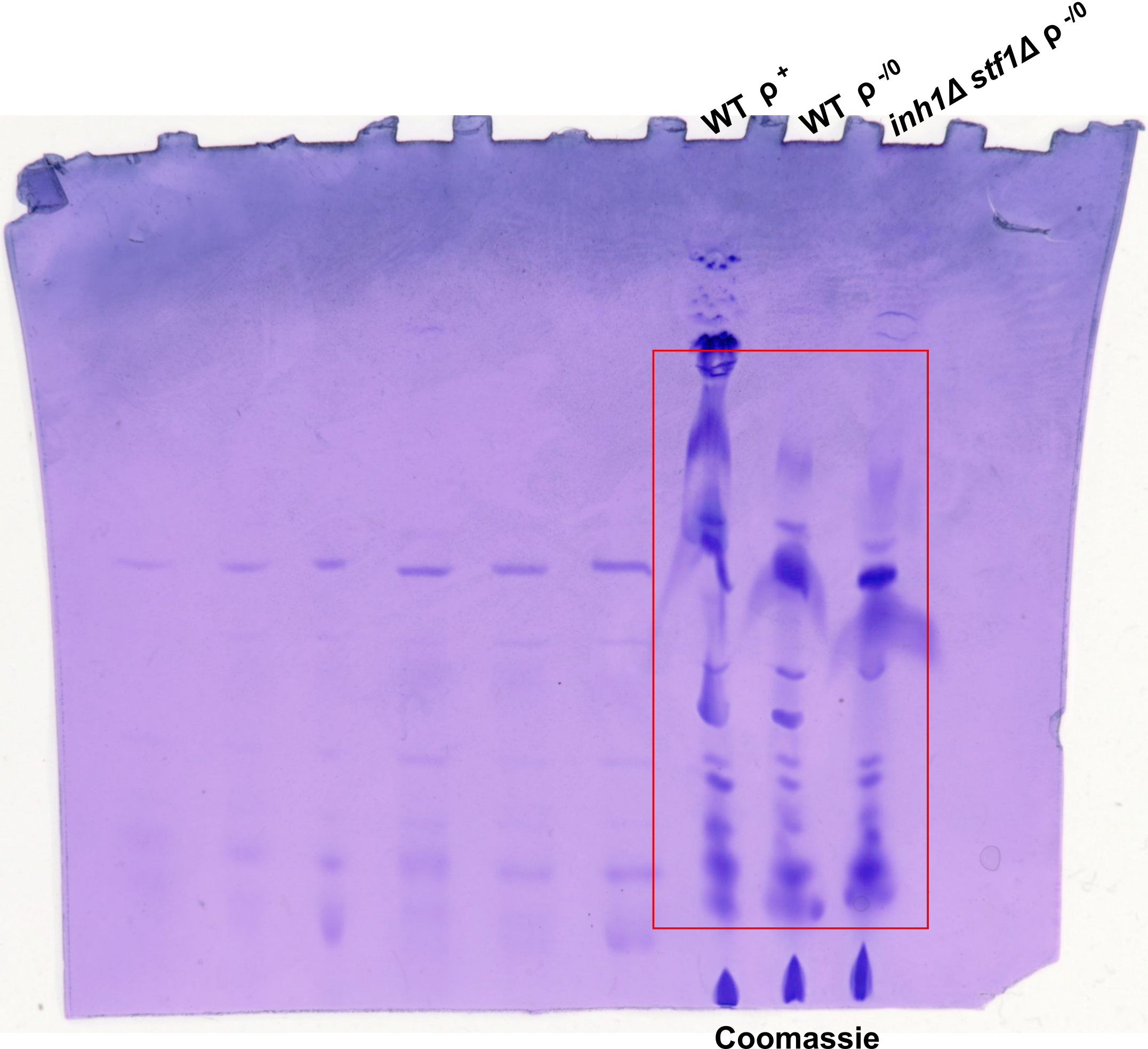

Supplement: Supplementary file 8 — Source data Fig. 6 [file 44319_2025_430_MOESM8_ESM.zip › Figure 6/6F/Coomassie.tiff]

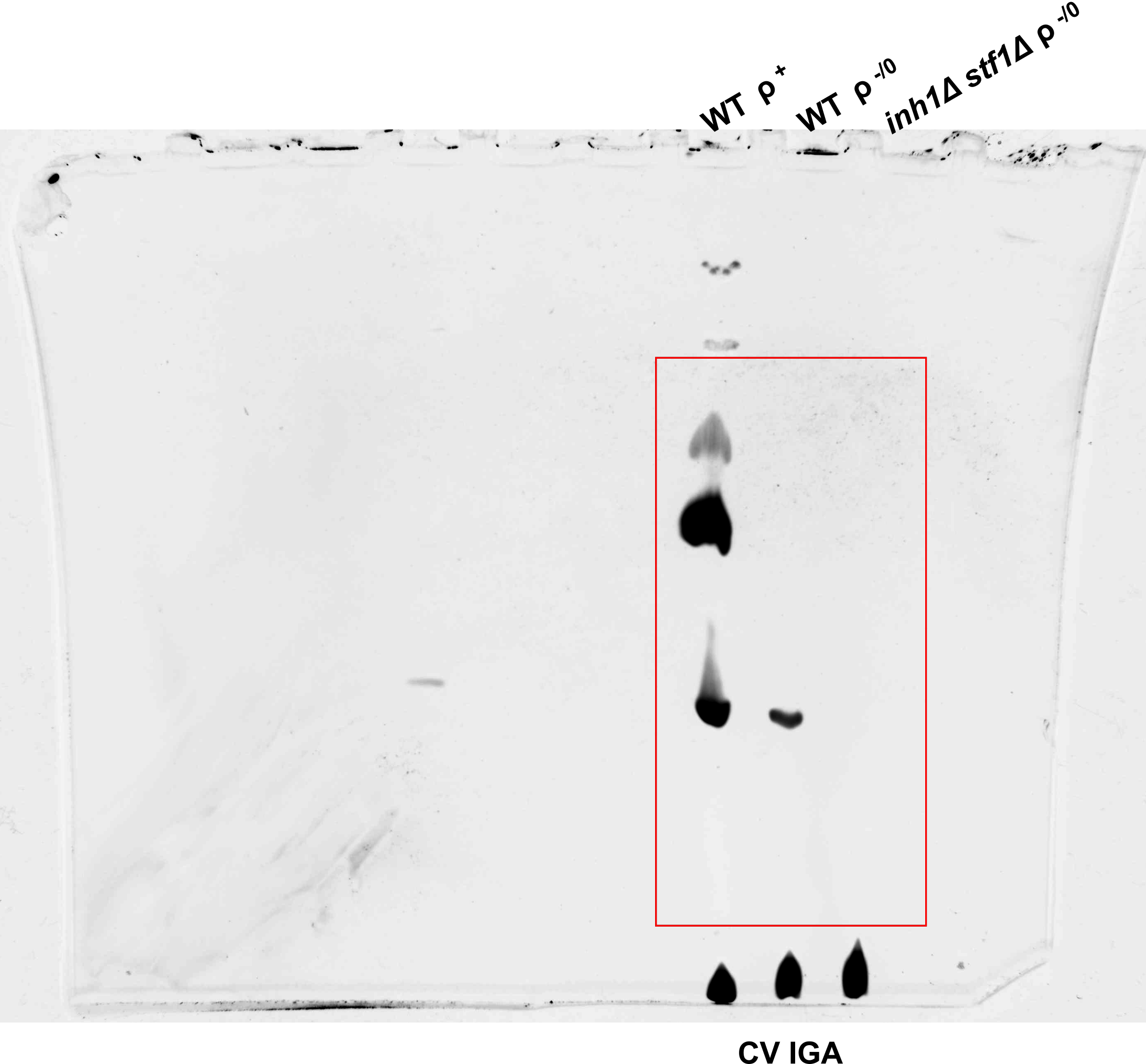

Supplement: Supplementary file 8 — Source data Fig. 6 [file 44319_2025_430_MOESM8_ESM.zip › Figure 6/6F/CV in gel activity.tiff]
