# Supplementary material for: An alternative mechanism by which If1 prevents ATP hydrolysis by the ATP synthase subcomplex in S. cerevisiae
Source: EMBO Rep. 2025 Jun 9;26(13):3305–26. doi: 10.1038/s44319-025-00430-8 (PMC12238618; doi:10.1038/s44319-025-00430-8)
Supplement: Supplementary file 9 — Expanded View Figures [file 44319_2025_430_MOESM9_ESM.pdf]

Expanded View Figures

Figure EV1. If1/Stf1 are required to maintain the F_1F_O-ATP synthase-free F_1 subcomplex level and activity.

BN-PAGE (3–12%) (**A**) and densitometric analysis (**B**) performed with total cell extracts, from WT, *inh1Δ stf1Δ*, and *atp18Δ* grown under glycerol 2% rich medium. Cell extracts were solubilized with increasing digitonin-to-protein ratio ranging from 0.5 to 6 g/g protein. The ATP synthase assemblies (O: oligomers; V: monomers; F_1: free F_1 subcomplex) were revealed by F_1F_O-ATP synthase (CV) hydrolytic in-gel activity (IGA). ($n = 3$, from left to right $*p = 0.0171$, $**p = 0.0081$, $**p = 0.0046$, $*p = 0.0441$, $**p = 0.0045$, $***p = 0.0007$, two-way ANOVA, error bars ± SEM). (**C**) Measurement of the ATP hydrolysis flux performed on purified mitochondria from total cell extracts from WT (black bars) and *inh1Δ* (red bars) grown on lactate 2% rich medium by monitoring the ATP induced phosphate production flux over several minutes. Experiments were performed at pH 9.0 (inactive inhibitors) and pH 6.4 (active inhibitors). ($n \geq 7$ independent experiments, $***p < 0.0001$, unpaired t-test, error bars ± SEM). (**D**) Determination of doubling time (hours) of WT (black bars) and *inh1Δ stf1Δ* (red bars) grown on different fermentable (glucose 0.5%, galactose 2%) and non-fermentable (glycerol 2%, lactate 2%) culture-rich media supplemented or not with CCCP (1, 1.5 or 2 µM), following the optical density of the culture at 550 nm. *nd*: too slow to determine the doubling time ($n = 3$ independent experiments, $**p = 0.0017$, two-way ANOVA, error bars ± SEM). (**E**) BN-PAGE (3–12%) performed with total cell extracts from WT grown on different fermentable (glucose 0.5%, galactose 2%) and non-fermentable (glycerol 2%, lactate 2%) culture-rich media. Cell extracts were solubilized with digitonin at a digitonin-to-protein ratio of 1.5 g/g protein. 150 µg of protein were loaded for glucose or galactose condition and 100 µg of protein were loaded for glycerol or lactate conditions. The F_1F_O-ATP synthase assemblies were revealed by F_1F_O-ATP synthase (CV) hydrolytic in-gel activity (IGA). (Representative of $n = 2$ independent experiments). (**F**) Western blot (left) and densitometric analysis (right) of the relative abundance of If1 and Stf1, using Pgk1 as loading control. Denaturing electrophoresis was performed with total cell extracts grown on glycerol 2% rich medium supplemented or not with CCCP. The Coomassie blue staining on the upper panel demonstrates the equal loading of tagged If1 and Stf1 produced in vitro and used for the relative quantification of inhibitors (* signal remaining from anti-STF1 antibody). The Coomassie staining of the purified peptides is also presented in Figs. 3B and 5A. ($n = 3$ independent experiments, unpaired t-test, error bars ± SEM). (**G**) Densitometric analysis of drop test performed on WT (black) and *atp18Δ* (orange) mutant grown on glycerol 2% culture minimum medium. ($n = 3$ independent experiments, from left to right $***p < 0.0003$, $***p < 0.0004$, $*p = 0.0227$, two-way ANOVA, error bars ± SEM). (**H**) Densitometric analysis of drop test performed on WT (black) and *cdc19Δ (green)* thermosensitive (*ts*) mutant (37°) mutant grown on glycerol 2% culture minimum medium. ($n = 3$ independent experiments, from left to right $***p < 0.0001$, $***p = 0.0003$, two-way ANOVA, error bars ± SEM). (**I**) Densitometric analysis of drop test performed on WT (black) and *cdc19Δ* (green) thermosensitive (*ts*) mutant (37°) mutant grown on lactate 2% culture minimum medium. ($n = 3$ independent experiments, from left to right $***p < 0.0004$, $***p < 0.0001$, $**p = 0.0088$, two-way ANOVA, error bars ± SEM).

 © The Author(s)

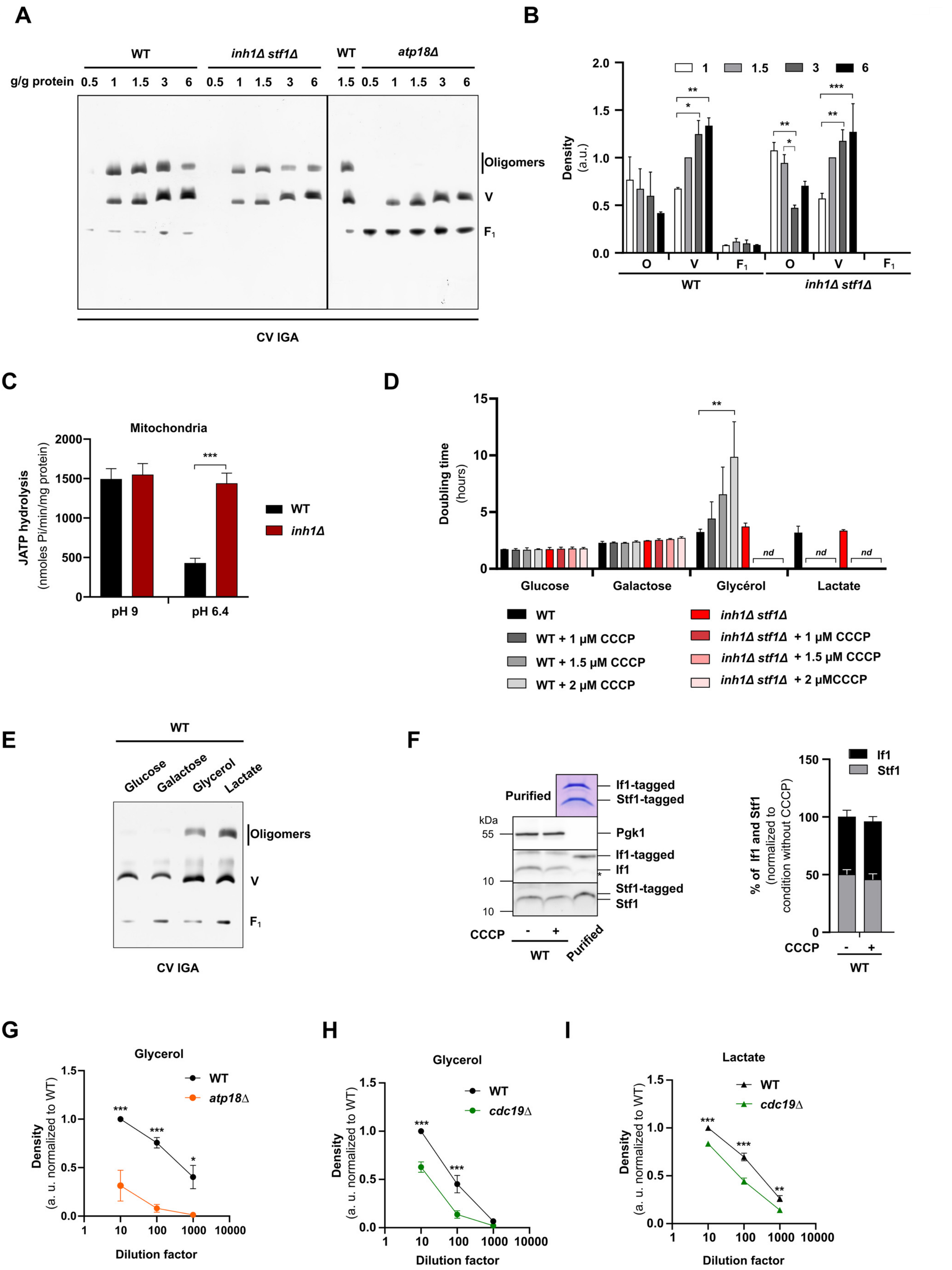
A
WT
inh1Δ stf1Δ
WT
atp18Δ
g/g protein 0.5 1 1.5 3 6 0.5 1 1.5 3 6 1.5 0.5 1 1.5 3 6
Oligomers
V
F1
CV IGA
B
Density (a.u.)
1 1.5 3 6
O V F1 O V F1
WT inh1Δ stf1Δ
C
Mitochondria
JATP hydrolysis (nmoles Pi/min/mg protein)
WT
inh1Δ
pH 9 pH 6.4
D
Doubling time (hours)
nd
Glucose Galactose Glycérol Lactate
WT
WT + 1 µM CCCP
WT + 1.5 µM CCCP
WT + 2 µM CCCP
inh1Δ stf1Δ
inh1Δ stf1Δ + 1 µM CCCP
inh1Δ stf1Δ + 1.5 µM CCCP
inh1Δ stf1Δ + 2 µMCCCP
E
WT
Glucose Galactose Glycerol Lactate
Oligomers
V
F1
CV IGA
F
Purified
If1-tagged
Stf1-tagged
kDa
55
Pgk1
If1-tagged
If1
10
Stf1-tagged
Stf1
10
CCCP - +
WT Purified
% of If1 and Stf1 (normalized to condition without CCCP)
If1
Stf1
CCCP - +
WT
G
Glycerol
Density (a. u. normalized to WT)
WT
atp18Δ
Dilution factor
H
Glycerol
WT
cdc19Δ
Dilution factor
I
Lactate
WT
cdc19Δ
Dilution factor

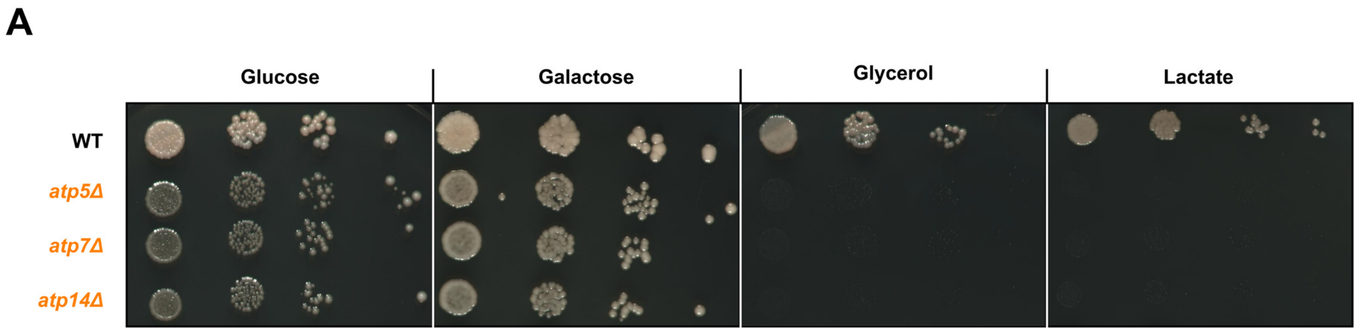

Figure EV2. The growth phenotype of F_1F_O-ATP synthase-deficient strains on various carbon sources.

(**A**) Drop test performed on WT, *atp5*Δ, atp7Δ, and *atp14*Δ mutant grown on different fermentable (glucose 0.5%, galactose 2%) or non-fermentable (glycerol 2%, lactate 2%) culture minimum media. (Representative of $n = 3$ independent experiments).

 © The Author(s)
